# Synthesis of triazolo- and tetrazolo-fused 1,4-benzodiazepines via one-pot Ugi–azide and Cu-free click reactions

**DOI:** 10.3762/bjoc.21.167

**Published:** 2025-10-17

**Authors:** Xiaoming Ma, Zijie Gao, Jiawei Niu, Wentao Shao, Shenghu Yan, Sai Zhang, Wei Zhang

**Affiliations:** 1 School of Pharmacy, Changzhou University, 1 Gehu Road, Changzhou 213164, China; 2 Department of Chemistry and Center for Green Chemistry, University of Massachusetts Boston, 100 Morrissey Blvd, Boston, MA 02125, USA

**Keywords:** benzodiazepine, click reaction, multicomponent reaction, one-pot, piperazinone, polycyclic, triazole, tetrazole, Ugi–azide reaction

## Abstract

A one-pot Ugi–azide reaction followed by intramolecular Cu-free azide–alkyne cycloaddition generates a polycyclic scaffold **7** bearing polycyclic triazole, tetrazole, and benzodiazepine rings. This method could be extended for obtaining a more complicated scaffold **8** containing a piperazinone ring.

## Introduction

**Figure 1:**
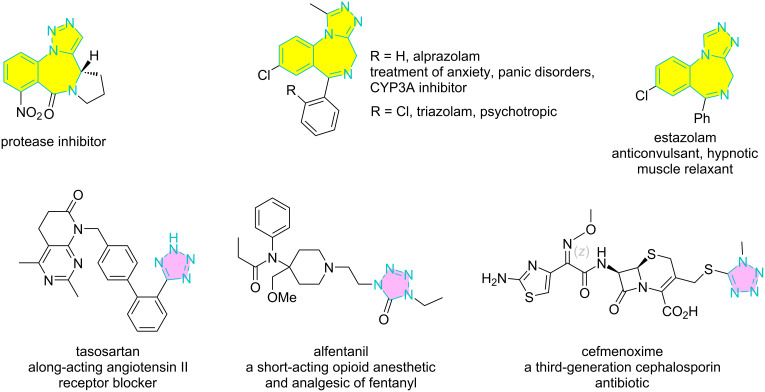
Some bioactive molecules bearing triazole, tetrazole, and 1,4-benzodiazepin rings.

Triazole, tetrazole, and benzodiazepine are privileged heterocyclic rings commonly found in drug molecules and functional materials [1–5]. For example, triazole-fused 1,4-benzodiazepins are protease inhibitors [6] and part of drug molecules such as alprazolam [7], estazolam [8], and triazolam [9] (Figure 1). Tetrazole-containing functional materials have been developed as photographic sensitizers, diagnostic contrast agents, and high-energy propellants [10–18]. Tetrazole is also found in several bioactive compounds [19–24], such as tasosartan, alfentanil, and cefmenoxime for the treatment of hypertension, anesthesia, and bacterial infections [4].

**Scheme 1:**
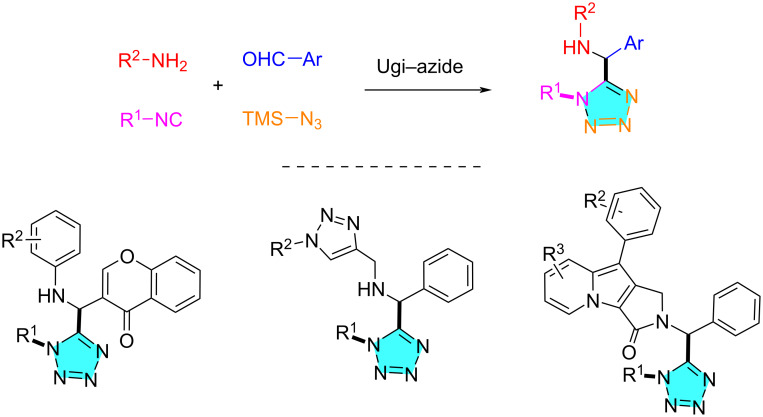
Ugi–azide reaction for the synthesis of 1,5-DS-T-containing heterocycles.

Among the reported methods for the synthesis of tetrazoles [25–28], the Ugi–azide reaction is a good approach for constructing 1,5-disubstituted-tetrazoles (1,5-DS-T) [29–31]. This scaffold can be subsequently linked to 1,2,3-triazole [32], 4*H*-chromen-4-one [33], pyrrolo[3,4-*b*]indolizine [34], and other heterocyclic scaffolds to obtain biologically interesting compounds (Scheme 1) [35–41].

**Scheme 2:**
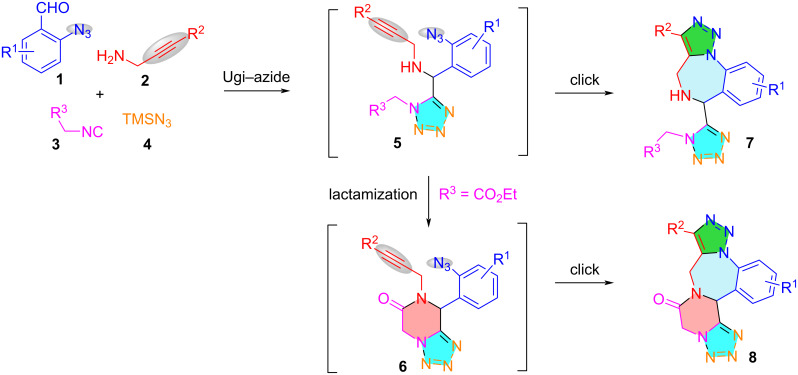
Proposed Ugi–azide-initiated synthesis of polyheterocyclic scaffolds **7** and **8**.

The development of methods for the synthesis of triazole, tetrazole, piperazinone, and 1,4-benzodiazepine motifs are attractive from both synthetic and medicinal chemistry considerations [42–45]. We herein propose a one-pot synthesis involving an Ugi–azide 4-component (4-CR) reaction followed by lactamization and azide–alkyne cycloaddition for assembling triazole-fused and tetrazole-tethered 1,4-benzodiazepines **7** and triazole-, tetrazole-, and piperazinone-fused 1,4-benzodiazepines **8** (Scheme 2). Functional groups including ester, azido, and alkynyl present in the starting materials are responsible for the post-Ugi transformations.

## Results and Discussion

**Scheme 3:**
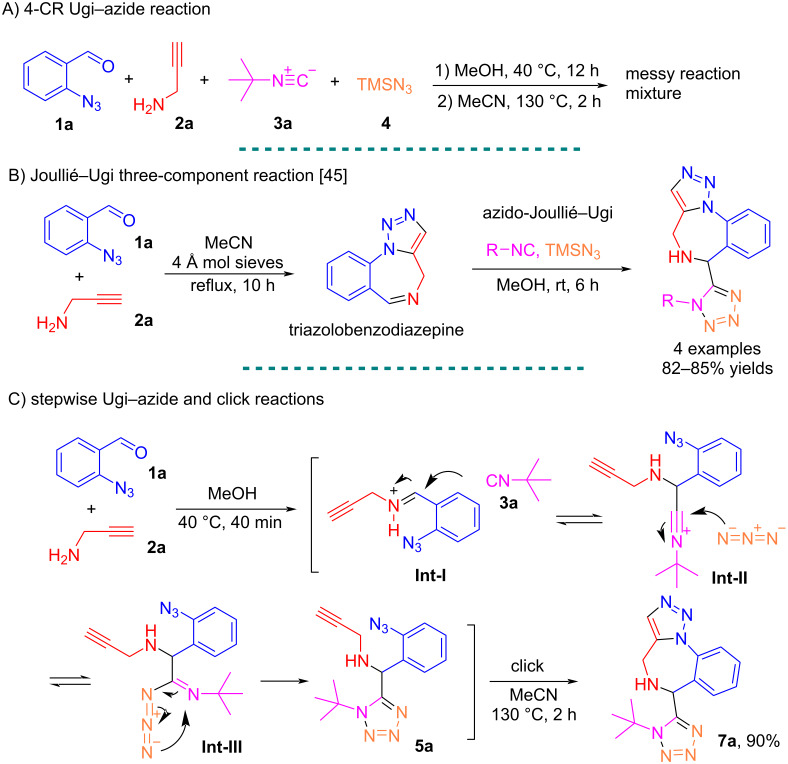
4-CR vs stepwise Ugi–azide reactions for the synthesis of **7a**.

We first attempted the Ugi–azide 4-CR at a 0.2 mmol scale with equal molar amounts of 2-azidobenzaldehyde (**1a**), propargylamine (**2a**), *tert*-butyl isocyanide (**3a**), and TMSN_3_ (**4**) in 2 mL of MeOH. After heating the reaction mixture at 40 °C for 12 h, the solvent was removed and changed to 2 mL of MeCN followed by heating at 130 °C for 2 h (Scheme 3A). However, no desired product was detected in the reaction mixture. The functional groups in four starting materials may not be compatible under the 4-CR conditions, in which propargylamine may undergo competitive reaction with 2-azidobenzaldehyde (**1a**) and TMSN_3_ (**4**). A literature search revealed that Shaabani’s group reported a reaction of 2-azidobenzaldehyde (**1a**) and propargylamine (**2a**) to obtain triazolobenzodiazepine which in turn can serve as a cyclic imine for a modified Joullié–Ugi 3-CR with isocyanide and trimethylsilyl azide (TMSN₃) in the synthesis of tetrazole-tethered triazolobenzodiazepines (Scheme 3B). We envisioned that the reaction conditions may need to be modified to introduce reactants in a sequential manner. Thus, we changed the reaction conditions by first reacting 0.2 mmol each of **1a** and **2a** in 2 mL MeOH at 40 °C for 40 min to form the Schiff base **Int-I** followed by the addition of 0.2 mmol each of **3a** and TMSN_3_ (**4**) to obtain the desired 1,5-DS-T **5a** as Ugi–azide adduct. After evaporation of the solvent MeOH, the residue was redissolved in 2 mL of MeCN and heated at 130 °C for 2 h in a sealed vial to give product **7a** in 90% yield after purification via Cu-free intramolecular click reaction (Scheme 3C) [46]. This Cu-free intramolecular cyclization provides key practical advantages over traditional copper-catalyzed azide–alkyne cycloaddition (CuAAC) reactions, including operational simplicity and the absence of metal contaminants, which is crucial for pharmaceutical applications.

**Scheme 4:**
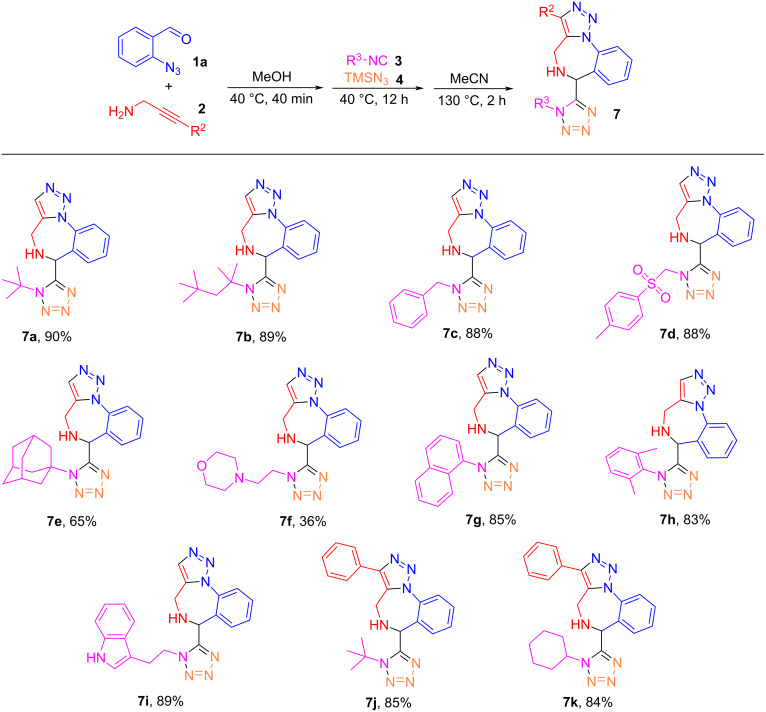
Synthesis of benzodiazepines **7a**–**k**. Reaction conditions: 1) 0.2 mmol each of 2-azidobenzaldehyde (**1a**) and 2-yn-1-amines **2** in MeOH (2 mL), 40 °C for 40 min; then add 0.2 mmol each of isocyanides **3** and TMSN_3_ (**4**), 40 °C for 12 h; 2) change solvent to MeCN (2 mL), 130 °C for 2 h.

After having identified suitable reaction conditions of the Ugi–azide and click reactions for the synthesis of triazole-fused and tetrazole-tethered benzodiazepine **7a**, we synthesized additional analogs by conducting the reaction of 2-azidobenzaldehyde (**1a**) with ten different isocyanides **3**, two 2-yn-1-amines (propargylamine (**2a)**, 3-phenylprop-2-yn-1-amine (**2b**)), and TMSN_3_ to give products **7a**–**k** in 36–90% yields (Scheme 4).

**Scheme 5:**
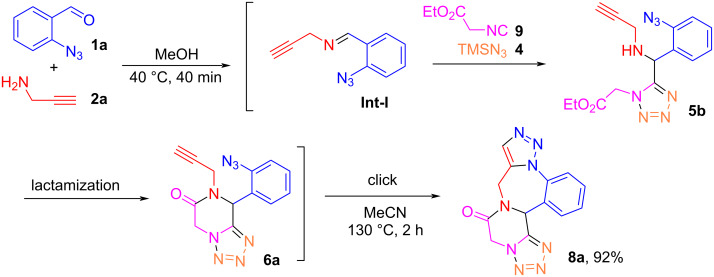
Synthesis of polycyclic compound **8a**.

As shown in Scheme 2, we also propose the synthesis of triazole-, tetrazole-, and piperazinone-fused 1,4-benzodiazepines **8**. For the synthesis of this unique polycyclic scaffold, 2-isocyanoacetate **9** played a critical role in the formation of the piperazinone ring. Thus, the reaction of 0.2 mmol each of **1a** and **2a** led to the formation of **Int-I** which then reacted with 0.2 mmol each of **9** and **4** to form 1,5-DS-T **5b** which consequently underwent lactamization to form **6a** followed by an intramolecular click reaction to afford highly condensed polycyclic product **8a** in 92% isolated yield (Scheme 5).

**Scheme 6:**
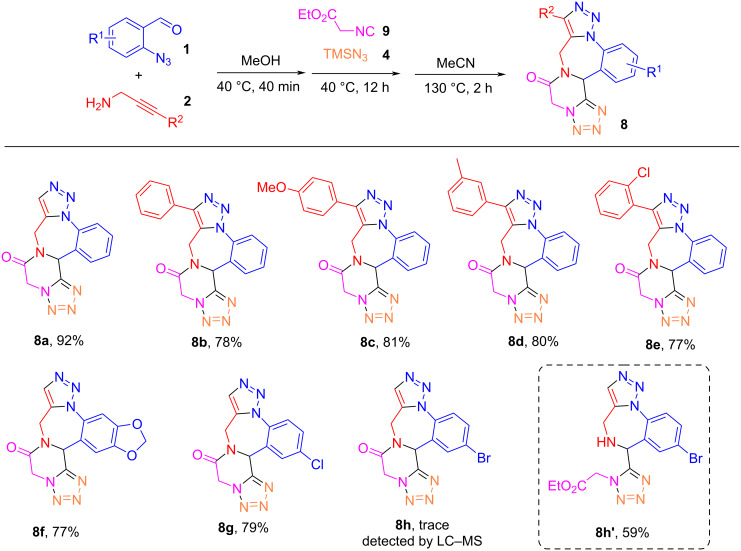
Synthesis of product analogs **8**. Reaction conditions: 1) 0.2 mmol each of 2-azidobenzaldehyde **(1a**) and 2-yn-1-amines **2** in MeOH (2 mL), 40 °C for 40 min; then addition of 0.2 mmol each of 2-isocyanoacetate (**9**) and TMSN_3_ (**4**), 40 °C for 12 h; 2) changing solvent to MeCN (2 mL), 130 °C for 2 h.

Next, the reaction scope was explored by reacting four different 2-azidobenzaldehydes **1** and five different 2-yn-1-amines **2** with 2-isocyanoacetate (**9**) and TMSN_3_ (**4**) to give products **8a**–**h** (Scheme 6). The reaction of five different 2-yn-1-amines **2** yielded products **8a**–**e** in 77–92% yields, which demonstrates a good tolerance of substituents R^2^ on 2-yn-1-amines **2**. The reaction of 6-azidobenzo[*d*][1,3]dioxol-5-carbaldehyde (**1b**) with propargylamine (**2a**), 2-isocyanoacetate (**9**), and TMSN_3_ (**4**) gave product **8f** in 77% yield. Azidobenzaldehyde bearing a Cl group gave product **8g** in 79% yield. However, the reaction of 2-azido-5-bromobenzaldehyde (**1d**) gave only a trace amount of product **8h**. Instead, compound **8h'**, an intermediate without lactamization, was isolated in 59% yield. It is likely that the bromo group on the phenyl ring interfered with the lactamization process.

**Scheme 7:**
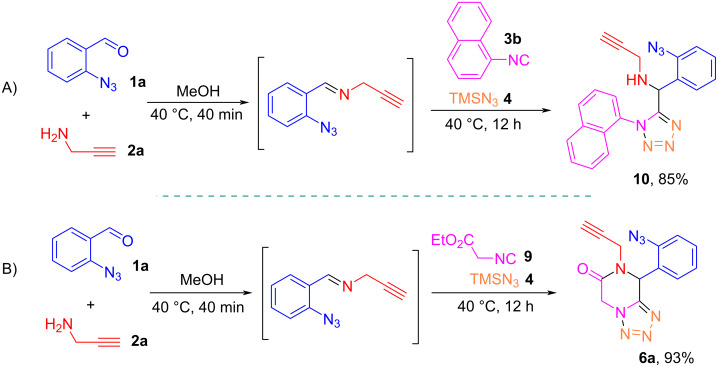
Control reactions to trap the Ugi–azide adduct.

Two control reactions were conducted to study the reaction process. The reaction of **1a**, **2a**, **3b**, and **4** at 40 °C afforded the Ugi–azide product **10** in 85% yield without formation of a triazole ring, which indicates that the intramolecular click reaction needs a higher temperature (Scheme 7A). The reaction involving the lactamization step was carried out using 2-isocyanoacetate (**9**) which gave the tetrazole-fused piperazinone **6a** in 93% yield, which also indicates that at this reaction temperature, lactamization could take place prior to the click reaction (Scheme 7B).

**Figure 2:**
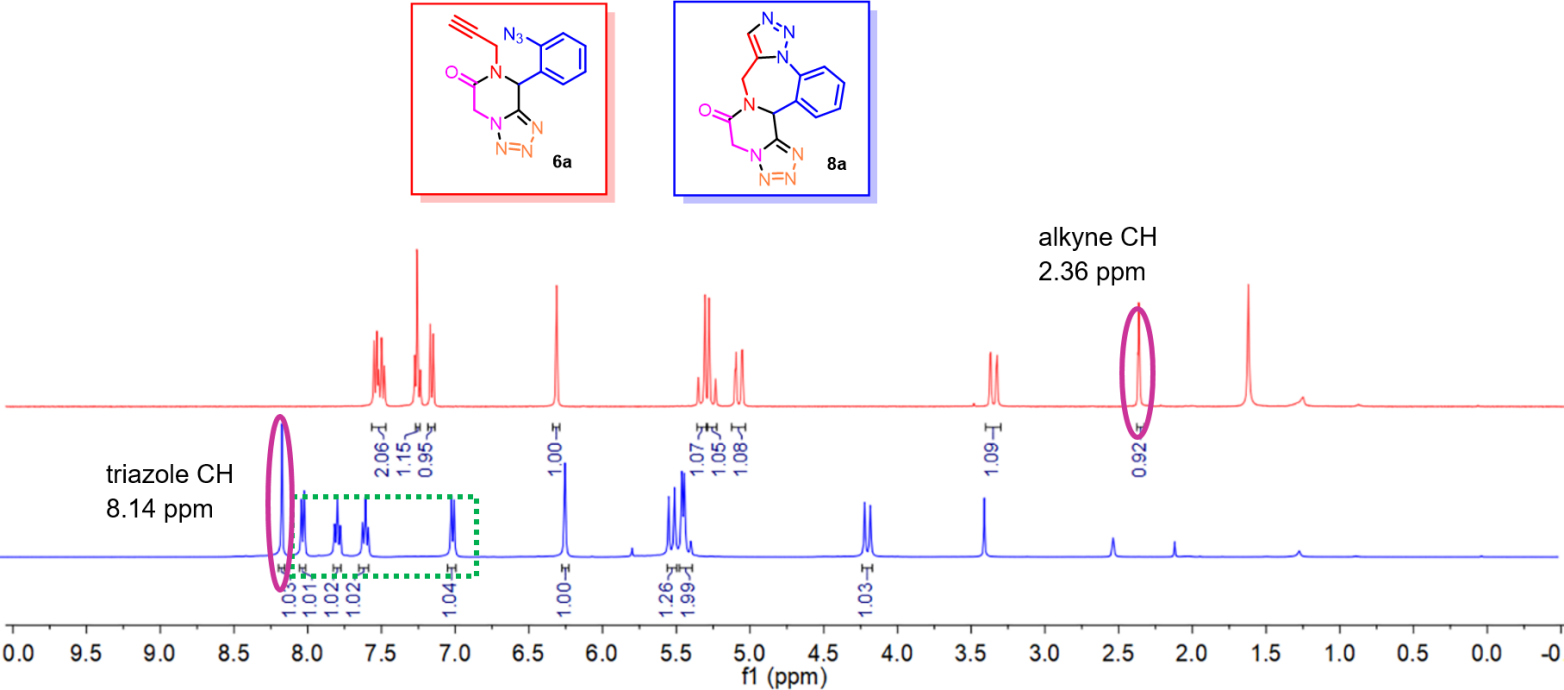
^1^H NMR spectra of compounds **6a** (red) and **8a** (blue).

Compounds **6a** and **8a** have same molecular weights, but their ^1^H NMR spectra are different (Figure 2). The most evident differences are the disappearance of the signal for the alkyne H at 2.36 ppm in the spectrum of **6a** and the appearance of a peak at 8.14 ppm for the proton on the triazole ring of **8a**. The aromatic protons in **8a** show two distinct doublets and two triplets which are slightly downfield-shifted as compared to the aromatic protons in compound **6a**. This observation reflects a rigid conformation of aromatic Hs after forming the 7-membered 1,4-diazepine ring in **8a**. In addition, the ^1^H-^1^H COSY and HSQC analysis of compound **8a** were conducted and the spectra are provided in Supporting Information File 1.

**Scheme 8:**
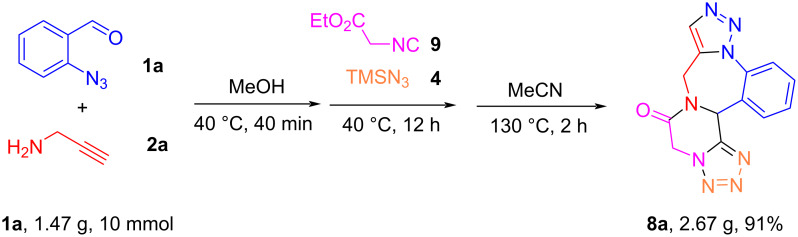
Gram-scale one-pot synthesis of **8a**.

To evaluate the scalability of this protocol, we performed the synthesis of diazepine **8a** on a gram scale with 10 mmol of **1a**, which led to the formation of product **8a** in 91% yield (Scheme 8).

## Conclusion

We have developed a new synthetic method to access triazole-fused and tetrazole-tethered benzodiazepines **7** via Ugi–azide/intramolecular click reaction under Cu-catalyst-free conditions. The 4-CR Ugi–azide reaction was modified to be a one-pot two-step reaction process to address functional group compatibility issues. By using 2-isocyanoacetate, the Ugi–azide adducts could undergo lactamization and lead to the formation of highly condensed 1,4-benzodiazepines **8** fused with triazole, tetrazole, and piperazinone rings. This is a new example of combining MCR and post-condensation modification as a one-pot synthesis to access novel heterocyclic scaffolds in a highly efficient manner. It was found that brominated azides do not undergo lactamization under the current conditions which presents an opportunity for further optimization of reaction conditions to expand the scope.

## Experimental

### General procedure for synthesis of analogs **7** and **8**

A solution of 2-azidobenzaldehyde **1** (0.2 mmol, 1 equiv) and propargylamine **2** (0.2 mmol, 1 equiv) in MeOH (2 mL) was heated at 40 °C for 40 min in a metal bath, followed by the addition of isocyanides **3** (0.2 mmol, 1 equiv) and TMSN_3_ (**4**, 0.2 mmol, 1 equiv) and further stirred at 40 °C for 12 h. Then, the reaction mixture was evaporated to remove MeOH solvent and the residue was redissolved in MeCN (2 mL) and heated at 130 °C for 2 h in a sealed vial. After the reaction had reached completion as monitored by TLC, the reaction mixture was concentrated in vacuo. Column chromatography on silica gel afforded products **7a**–**k** in 36–90% yields, **8a**–**g** in 77–92% yields.

**Note:** When 2-yn-1-amine hydrochlorides **2b**–**e** were employed in this one-pot reaction, Et_3_N (0.3 mol, 1.5 equiv) was added into the vessel at the initial stage.

## Supporting Information

File 1 Experimental section, characterization data and copies of spectra.

## Data Availability

All data that supports the findings of this study is available in the published article and/or the supporting information of this article.

## References

[1] Keri R S, Patil S A, Budagumpi S, Nagaraja B M (2015). Chem Biol Drug Des.

[2] Soyka M (2017). N Engl J Med.

[3] Sigel E, Ernst M (2018). Trends Pharmacol Sci.

[4] Neochoritis C G, Zhao T, Dömling A (2019). Chem Rev.

[5] Dhiman N, Kaur K, Jaitak V (2020). Bioorg Med Chem.

[6] Mohapatra D K, Maity P K, Shabab M, Khan M I (2009). Bioorg Med Chem Lett.

[7] Ye Z, Ding M, Wu Y, Li Y, Hua W, Zhang F (2018). Green Chem.

[8] Massah A R, Gharaghani S, Lordejani H A, Asakere N (2016). Med Chem Res.

[9] Fustero S, González J, Del Pozo C (2006). Molecules.

[10] Wei C-X, Bian M, Gong G-H (2015). Molecules.

[11] Frija L M T, Ismael A, Cristiano M L S (2010). Molecules.

[12] Myznikov L V, Hrabalek A, Koldobskii G I (2007). Chem Heterocycl Compd (N Y, NY, U S).

[13] Lv F, Liu Y, Zou J, Zhang D, Yao Z (2006). Dyes Pigm.

[14] Song W, Wang Y, Qu J, Madden M M, Lin Q (2008). Angew Chem, Int Ed.

[15] Shmatova O I, Nenajdenko V G (2013). J Org Chem.

[16] Dippold A A, Izsák D, Klapötke T M, Pflüger C (2016). Chem – Eur J.

[17] Fischer D, Klapötke T M, Stierstorfer J (2015). Angew Chem, Int Ed.

[18] Nasrollahzadeh M, Nezafat Z, Bidgoli N S S, Shafiei N (2021). Mol Catal.

[19] Muraglia E, Kinzel O D, Laufer R, Miller M D, Moyer G, Munshi V, Orvieto F, Palumbi M C, Pescatore G, Rowley M (2006). Bioorg Med Chem Lett.

[20] Kambe T, Correia B E, Niphakis M J, Cravatt B F (2014). J Am Chem Soc.

[21] Chauhan K, Singh P, Kumar V, Shukla P K, Siddiqi M I, Chauhan P M S (2014). Eur J Med Chem.

[22] Yuan Y, Li M, Apostolopoulos V, Matsoukas J, Wolf W M, Blaskovich M A T, Bojarska J, Ziora Z M (2024). Eur J Med Chem.

[23] Chowdhury M G, Kapoor S, Muthukumar V, Chatterjee D R, Shard A (2025). Bioorg Chem.

[24] Li G, Jiang B, Wang J-Y, Hao W-J (2023). Synlett.

[25] Savych O, Kuchkovska Y O, Bogolyubsky A V, Konovets A I, Gubina K E, Pipko S E, Zhemera A V, Grishchenko A V, Khomenko D N, Brovarets V S (2019). ACS Comb Sci.

[26] Soni A, Kumar P, Tomar V, Joshi R K, Nemiwal M (2023). Synth Commun.

[27] Moraes A M, da Silva T L, de Mattos M C S (2023). Curr Org Chem.

[28] Pharande S G, Rentería-Gómez M A, Gámez-Montaño R (2018). New J Chem.

[29] Medda F, Martinez-Ariza G, Hulme C (2015). Tetrahedron Lett.

[30] Haldar S, Saha S, Mandal S, Jana C K (2018). Green Chem.

[31] Mohammadkhani L, Heravi M M (2020). Mol Diversity.

[32] Aguilar-Morales C M, Araujo-Huitrado J G, López-Hernández Y, Contreras-Celedón C, Islas-Jácome A, Granados-López A J, Solorio-Alvarado C R, López J A, Chacón-García L, Cortés-García C J (2021). Molecules.

[33] Cano P A, Islas-Jácome A, González-Marrero J, Yépez-Mulia L, Calzada F, Gámez-Montaño R (2014). Bioorg Med Chem.

[34] Niño‐Pantoja I, Gallardo‐Alfonzo A, Solis‐Santos M, Ordoñez M, Contreras‐Celedón C, Islas‐Jácome A, Chacón‐García L, Cortés‐García C J (2022). Eur J Org Chem.

[35] Zarganes‐Tzitzikas T, Patil P, Khoury K, Herdtweck E, Dömling A (2015). Eur J Org Chem.

[36] Saha D, Kharbanda A, Essien N, Zhang L, Cooper R, Basak D, Kendrick S, Frett B, Li H-y (2019). Org Chem Front.

[37] Lohmann N, Milovanović V, Piekarski D G, García Mancheño O (2022). Org Biomol Chem.

[38] Sun B-B, Liu K, Gao Q, Fang W, Lu S, Wang C-R, Yao C-Z, Cao H-Q, Yu J (2022). Nat Commun.

[39] Yang M-L, Zhao L, Chen H-R, Ding M-W (2023). J Org Chem.

[40] Niu J, Wang Y, Yan S, Zhang Y, Ma X, Zhang Q, Zhang W (2024). Beilstein J Org Chem.

[41] Zuo H-D, Chen X, Zhang Y, Liu J-W, Yan S-H, Li G, Wang J-Y (2024). Org Lett.

[42] Donald J R, Martin S F (2011). Org Lett.

[43] Nazeri M T, Farhid H, Mohammadian R, Shaabani A (2020). ACS Comb Sci.

[44] Aguilar-Morales C M, de Loera D, Contreras-Celedón C, Cortés-García C J, Chacón-García L (2019). Synth Commun.

[45] Nazeri M T, Ghasemi M, Ahmadi M, Shaabani A, Notash B (2023). J Org Chem.

[46] Ma X, Zhang X, Qiu W, Zhang W, Wan B, Evans J, Zhang W (2019). Molecules.

