# Supplementary material for: Synthesis of triazolo- and tetrazolo-fused 1,4-benzodiazepines via one-pot Ugi–azide and Cu-free click reactions
Source: Beilstein J Org Chem. 2025 Oct 17;21:2202–10. doi: 10.3762/bjoc.21.167 (PMC12536470; doi:10.3762/bjoc.21.167)
Supplement: File 1 — Experimental section, characterization data and copies of spectra. [file Beilstein_J_Org_Chem-21-2202-s001.pdf]

BEILSTEIN JOURNAL OF ORGANIC CHEMISTRY

Supporting Information

for

Synthesis of triazolo- and tetrazolo-fused 1,4-benzodiazepines via one-pot Ugi–azide and Cu-free click reactions

Xiaoming Ma, Zijie Gao, Jiawei Niu, Wentao Shao, Shenghu Yan, Sai Zhang and Wei Zhang

Beilstein J. Org. Chem. **2025,** *21,* 2202–2210. doi:10.3762/bjoc.21.167

Experimental section, characterization data and copies of spectra

License and Terms: This is a supporting information file under the terms of the Creative Commons Attribution License (https://creativecommons.org/licenses/by/4.0). Please note that the reuse, redistribution and reproduction in particular requires that the author(s) and source are credited and that individual graphics may be subject to special legal provisions.
The license is subject to the *Beilstein Journal of Organic Chemistry* terms and conditions: (https://www.beilstein-journals.org/bjoc/terms)

Table of contents

1. General information S2
2. General procedure for synthesis of analogs **7** and **8** (GP) S2
3. General procedure for the synthesis of propargylic amine hydrochloride salts S3
4. Control experiments S4
5. Analytic data of products S5
6. NMR spectra of compounds **7** and **8** S13
7. H-H COSY and HSQC spectra of compound **8a** S34

1. General information

^{1}H (400 MHz or 300 MHz) and ^{13}C NMR spectra (101 MHz or 75 MHz) were recorded on a Bruker DPX 400 NMR spectrometer or a Bruker Avance III 300 NMR spectrometer. Data are reported as follows: chemical shift (multiplicity, coupling constants, number of protons). Coupling constants were quoted to the nearest 0.1 Hz, and multiplicity was reported according to the following convention: s, singlet; d, doublet; t, triplet; q, quartet; m, multiplet; br, broad. HRMS was performed on an Agilent 6540 Q-TOF mass spectrometer (ESI). Flash column chromatography was performed using silica gel (200–300 mesh). Chemicals and solvents, unless otherwise noted, were purchased from Energy Chemical, Adamas or BidePharm and used without further purification. Melting points were measured with JiaHang JH30 apparatus and are uncorrected.

2. General procedure for synthesis of analogs 7 and 8 (GP)

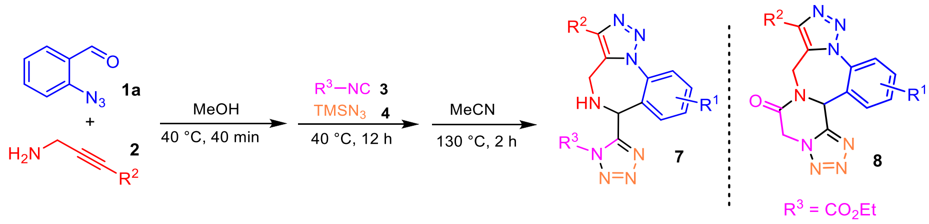

A solution of 2-azidobenzaldehyde **1** (0.2 mmol, 1 equiv) and propargylamine **2** (0.2 mmol, 1 equiv) in MeOH (2 mL) was heated at 40 °C for 40 min in a metal bath, then followed by the addition of isocyanides **3** (0.2 mmol, 1 equiv) and $TMSN_3$ (**4**, 0.2 mmol, 1 equiv), and stirred at 40 °C for 12 h. Next, the reaction mixture was evaporated to remove MeOH solvent, the residue redissolved in MeCN (2 mL) and heated at 130 °C for 2 h in a sealed vial. After the reaction had reached completion as monitored by TLC, the reaction mixture was concentrated in vacuo, and isolated by chromatography column on silica gel to afford products **7a**–**k** in 36–90% yields, **8a**–**g** in 77–92% yields.

Note: When 2-yn-1-amine hydrocholorides **2b**–**e** were employed in this one-pot reaction, Et_3N (0.3 mol, 1.5 equiv) was added into the vessel at the initiate stage.

3. General procedure for the synthesis of propargylic amine hydrochloride salts [1]

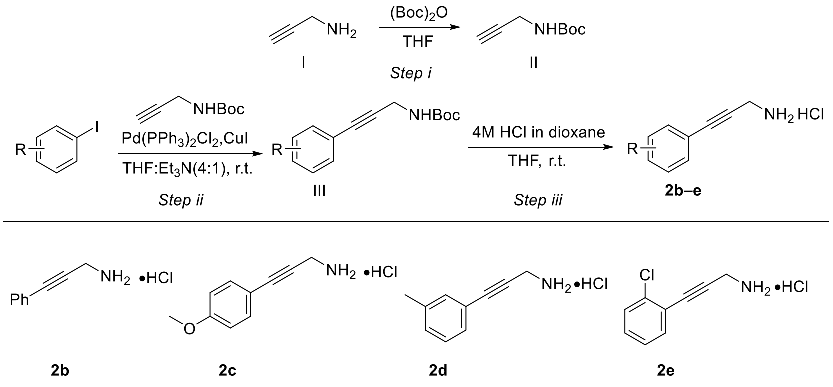

The aryl-substituted propargylic amines **2b**–**d** were prepared according to the literature precedure[1] in three steps with the synthesis of compound **2b** as representative example below.

Step i. Synthesis of compound II.

To a solution of terminal propargylamine **I** (10.0 g, 0.182 mol) in THF (300 mL) was added $(Boc)_2O$ (39.7 g, 0.182 mmol) slowly at 0 °C. After the addition, the reaction mixture was allowed to warm to room temperature and stirred for 2 h. Upon completion, the solution was concentrated under reduced pressure, and then the residue was purified by column chromatography (petroleum ether/ethyl acetate 10:1) to afford compound **II** (27.4 g, 97% yield) as a white solid.

Step ii. Synthesis of compound III.

To a mixture of compound **II** (10.0 g, 64 mmol), CuI (0.486 g, 2.56 mmol) and $PdCl_2(PPh_3)_2$ (0.898 g, 1.28 mmol) in THF/Et_3N (120 mL/30 mL) was added iodobenzene (14.4 g, 70.4 mmol). The mixture was stirred under N_2 atmosphere at room temperature overnight. Upon completion, the reaction was quenched with saturated NH_4Cl aqueous solution (100 mL) and extracted with EtOAc (100 mL × 3). The combined organic layers were washed with brine, dried over anhydrous Na_2SO_4, filtered, concentrated via rotary evaporation, and purified by column chromatography on silica gel (petroleum ether/ethyl acetate 30:1) to give compound **III** (8.73 g, 59%) as a yellow solid.

Step iii. Synthesis of compound 2b–e.

To a solution of **III** (2.0 g, 8.65 mmol) in THF (4 mL) was added 4.0 M HCl dioxane solution (8.7 mL, 34.8 mmol) at room temperature, and the reaction mixture was stirred overnight. The solution was diluted with Et_2O (20 mL) and a white precipitate appeared. The organic layer was removed by decantation, and the precipitate was washed with Et_2O three times. The solid compound was dried under vacuum to give the corresponding propargylic amine hydrochloride salts **2b**–**e**.

References

Ji, P.; Liu, X,; Xu, J.; Zhang, X.; Guo, J.; Chen W.-W.; Zhao, B. Direct Asymmetric α-C−H Addition of *N*-unprotected Propargylic Amines to Trifluoromethyl Ketones by Carbonyl Catalysis. *Angew. Chem. Int. Ed.* **2022**, *61*, e202206111. https://doi.org/10.1002/anie.202206111.

4. Control experiments

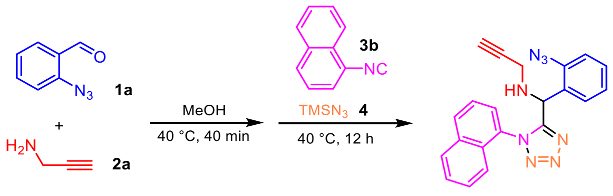

10, 85%

A solution of 2-azidobenzaldehyde (**1a**, 0.2 mmol, 1 equiv) and propargylamine (**2a**, 0.2 mmol, 1 equiv) in MeOH (2 mL) was heated at 40 °C for 40 min in a metal bath, followed by the addition of 1-naphthyl isocyanide (**3b**, 0.2 mmol, 1 equiv) and $TMSN_3$ (**4**, 0.2 mmol, 1 equiv), and the mixture was stirred at 40 °C for 12 h. After the Ugi–azide reaction had reached completion as monitored by TLC, the reaction mixture was concentrated in vacuo, and the residue separated by column chromatography (petroleum ether/ethyl acetate 1:1) on silica gel to afford product **10** in 85% yield (65 mg) as a white solid.

***N*-((2-Azidophenyl)(1-(naphthalen-1-yl)-1*H*-tetrazol-5-yl)methyl)prop-2-yn-1-amine (10)**

Melting point: 122–124 °C. ^{1}H NMR (400 MHz, DMSO-d_6) δ 8.24 (d, J = 2.0 Hz, 1H), 8.19 (d, J = 8.8 Hz, 1H), 8.14–8.03 (m, 2H), 7.75–7.67 (m, 3H), 7.64 (dd, J = 7.6, 1.6 Hz, 1H), 7.35 (td, J = 7.6, 1.6 Hz, 1H), 7.20 (td, J = 7.6, 1.6 Hz, 1H), 7.13 (dd, J = 8.0, 1.2 Hz, 1H), 5.59 (d, J = 7.2 Hz, 1H), 3.66 (q, J = 6.2 Hz, 1H), 3.48–3.38 (m, 1H), 3.28–3.18 (m, 1H), 3.01 (t, J = 2.6 Hz, 1H). ^{13}C NMR (101 MHz, DMSO-d_6) δ 156.6, 137.7, 133.6, 132.8, 131.2, 130.1, 130.0, 129.2, 129.0, 128.9, 128.4, 128.1, 125.6, 125.3, 123.4, 119.0, 81.9, 75.1, 49.0, 35.8. HRMS (ESI-TOF) m/z calcd for $C_{21}H_{16}N_8Na^+$ $[M+Na]^+$ 403.1390, found 403.1390.

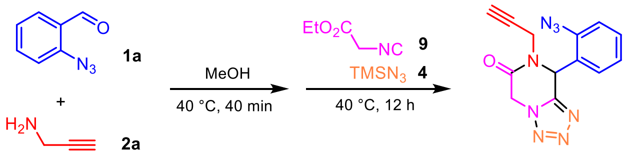

6a, 93%

A solution of 2-azidobenzaldehyde (**1a**, 0.2 mmol, 1 equiv) and propargylamine (**2a**, 0.2 mmol, 1 equiv) in MeOH (2 mL) was heated at 40 °C for 40 min in a metal bath, followed by the addition of 2-isocyanoacetate **9** (0.2 mmol, 1 equiv) and $TMSN_3$ (**4**, 0.2 mmol, 1 equiv), and the mixture was stirred at 40 °C for 12 h. After the Ugi–azide reaction had reached completion as monitored by TLC, the reaction mixture was concentrated in vacuo, and the residue separated by column chromatography (petroleum ether/ethyl acetate 1:1) on silica gel to afford product **6a** in 93% yield (55 mg) as a white solid.

8-(2-Azidophenyl)-7-(prop-2-yn-1-yl)-7,8-dihydrotetrazolo[1,5-*a*]pyrazin-6(5*H*)-one (6a)

Melting point: 130–132 °C. ^{1}H NMR (400 MHz, $CDCl_3$) δ 7.56–7.47 (m, 2H), 7.28–7.23 (m, 1H), 7.16 (d, J = 8.0 Hz, 1H), 6.31 (s, 1H), 5.33 (dd, J = 18.0, 1.6 Hz, 1H), 5.26 (dd, J = 18.0, 1.6 Hz, 1H), 5.08 (dd, J = 17.6, 2.4 Hz, 1H), 3.35 (dd, J = 17.6, 2.4 Hz, 1H), 2.36 (t, J = 2.6 Hz, 1H). ^{13}C NMR (101 MHz, $CDCl_3$) δ 168.8, 154.6, 149.6, 136.9, 136.4, 136.0, 126.5, 122.9, 120.1, 52.3, 27.9, 26.1, 25.2, 16.8. HRMS (ESI-TOF) m/z calcd for $C_{13}H_{10}N_8NaO^+$ $[M+Na]^+$ 317.0870, found 317.0877.

5. Analytic data of products

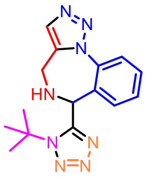

7a

6-(1-(*tert*-Butyl)-1*H*-tetrazol-5-yl)-5,6-dihydro-4*H*-benzo[*f*][1,2,3]triazolo[1,5-*a*][1,4]diazepine (7a)

According to GP and purified by column chromatography (petroleum ether/ethyl acetate 1:2) to afford a white solid in 90% yield (56 mg). Melting point: 156–158 °C. ^{1}H NMR (400 MHz, $CDCl_3$) δ 7.94–7.85 (m, 1H), 7.84–7.73 (m, 1H), 7.61–7.49 (m, 1H), 7.43–7.29 (m, 1H), 6.70–6.60 (m, 1H), 5.32–5.16 (m, 1H), 4.40–4.32 (m, 1H), 3.90–3.78 (m, 1H), 3.31–3.16 (m, 1H), 1.42–1.32 (m, 9H). ^{13}C NMR (101 MHz, $CDCl_3$) δ 152.7, 135.5, 134.1, 132.9, 130.5, 130.3, 129.8, 129.8, 128.0, 123.8, 62.1, 52.7, 37.8, 29.8, 29.7. HRMS (ESI-TOF) m/z calcd for $C_{15}H_{18}N_8Na^+$ $[M+Na]^+$ 333.1547, found 333.1545.

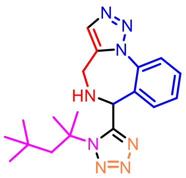

7b

6-(1-(2,4,4-Trimethylpentan-2-yl)-1*H*-tetrazol-5-yl)-5,6-dihydro-4*H*-benzo[*f*][1,2,3]triazolo[1,5-*a*][1,4]diazepine (7b)

According to GP and purified by column chromatography (petroleum ether/ethyl acetate 1:2) to afford a yellow solid in 89% yield (65 mg). Melting point:153–155 °C. ^{1}H NMR (400 MHz, DMSO-d_6) δ 8.08 (dd, J = 8.0, 1.2 Hz, 1H), 7.82 (s, 1H), 7.62 (td, J = 7.6, 1.2 Hz, 1H), 7.46 (td, J = 7.6, 1.2 Hz, 1H), 7.11 (dd, J = 8.0, 1.2 Hz, 1H), 5.62 (d, J = 8.4 Hz, 1H), 4.19 (dd, J = 14.8, 3.2 Hz, 1H), 4.07–3.98 (m, 1H), 3.79 (dd, J = 14.8, 6.4 Hz, 1H), 1.90 (d, J = 15.2 Hz, 1H), 1.77 (d, J = 15.2 Hz, 1H), 1.74 (s, 3H), 1.54 (s, 3H), 0.64 (s, 9H). ^{13}C NMR (101 MHz, DMSO-d_6) δ 153.9, 136.2, 135.7, 132.0, 130.5, 129.7, 129.4, 128.8, 123.0, 65.2, 53.7, 52.1, 37.5, 31.1, 30.2, 29.4, 29.3. HRMS (ESI-TOF) m/z calcd for $C_{19}H_{26}N_8Na^+$ $[M+Na]^+$ 389.2173, found 389.2167.

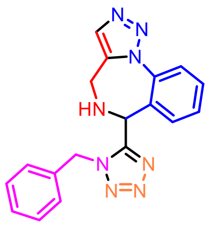

7c

6-(1-Benzyl-1*H*-tetrazol-5-yl)-5,6-dihydro-4*H*-benzo[*f*][1,2,3]triazolo[1,5-*a*][1,4]diazepine (7c)

According to GP and purified by column chromatography (petroleum ether/ethyl acetate 1:2) to afford a white solid in 88% yield (61 mg). Melting point: 151–153 °C. ^{1}H NMR (400 MHz, DMSO-d_6) δ 7.93 (d, J = 8.0 Hz, 1H), 7.63 (td, J = 7.6, 1.2 Hz, 1H), 7.57 (s, 1H), 7.45 (t, J = 7.6 Hz, 1H), 7.39–7.30 (m, 3H), 7.27–

7.14 (m, 3H), 5.85–5.65 (m, 3H), 4.23 (q, *J* = 5.4 Hz, 1H), 3.98–3.75 (m, 2H). ^{13}C NMR (101 MHz, DMSO-d_6) δ 154.8, 135.8, 134.8, 134.7, 131.8, 130.1, 129.9, 129.8, 129.1, 128.6, 128.1, 128.0, 123.2, 53.3, 50.7, 36.8. HRMS (ESI-TOF) m/z calcd for $C_{18}H_{16}N_8Na^+$ [M+Na]$^+$ 367.1390, found 367.1387.

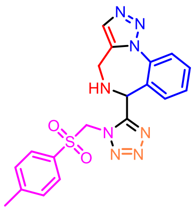

7d

6-(1-(Tosylmethyl)-1*H*-tetrazol-5-yl)-5,6-dihydro-4*H*-benzo[*f*][1,2,3]triazolo[1,5-*a*][1,4]diazepine (7d)

According to GP and purified by column chromatography (petroleum ether/ethyl acetate 1:2) to afford a brown solid in 88% yield (74 mg). Melting point: 152–154 °C. ^{1}H NMR (400 MHz, DMSO-d_6) δ 7.95 (dd, *J* = 8.0, 1.2 Hz, 1H), 7.69 (td, *J* = 7.6, 1.6 Hz, 1H), 7.64–7.55 (m, 4H), 7.50–7.40 (m, 3H), 6.71 (d, *J* = 14.4 Hz, 1H), 6.50 (d, *J* = 14.4 Hz, 1H), 5.61 (d, *J* = 7.6 Hz, 1H), 4.38–4.30 (m, 1H), 4.05 (dd, *J* = 14.4, 2.4 Hz, 1H), 3.78 (dd, *J* = 14.0, 5.2 Hz, 1H), 2.43 (s, 3H). ^{13}C NMR (101 MHz, DMSO-d_6) δ 155.9, 146.1, 135.7, 134.1, 133.0, 132.1, 130.6, 130.2, 130.2, 129.6, 129.3, 128.7, 123.3, 79.2, 64.7, 53.9, 36.4, 21.2. HRMS (ESI-TOF) m/z calcd for $C_{19}H_{18}N_8SO_2Na^+$ [M+Na]$^+$ 445.1166, found 445.1160.

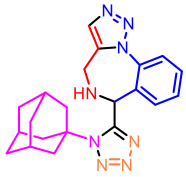

7e

6-(1-((3s,5s,7s)-Adamantan-1-yl)-1*H*-tetrazol-5-yl)-5,6-dihydro-4*H*-benzo[*f*][1,2,3]triazolo[1,5-*a*][1,4]diazepine (7e)

According to GP and purified by column chromatography (petroleum ether/ethyl acetate 1:2) to afford a white solid in 65% yield (51 mg). Melting point: 158–160 °C. ^{1}H NMR (400 MHz, DMSO-d_6) δ 8.12–8.07 (m, 1H), 7.77 (s, 1H), 7.62 (td, *J* = 7.6, 1.2 Hz, 1H), 7.46 (td, *J* = 7.6, 1.6 Hz, 1H), 7.08 (dd, *J* = 7.6, 1.6 Hz, 1H), 5.68 (d, *J* = 7.2 Hz, 1H), 4.17 (dd, *J* = 14.8, 3.6 Hz, 1H), 4.10–3.98 (m, 1H), 3.78 (dd, *J* = 14.8, 6.8 Hz, 1H), 2.28–2.20 (m, 3H), 2.18–2.09 (m, 6H), 1.69–1.59 (m, 6H). ^{13}C NMR (151 MHz, DMSO-d6) δ 154.4, 137.0, 136.2, 132.4, 131.4, 130.1, 130.0, 129.3, 123.4, 79.6, 63.2, 54.7, 41.5, 37.9, 35.4, 29.5. HRMS (ESI-TOF) m/z calcd for $C_{21}H_{24}N_8Na^+$ [M+Na]$^+$ 411.2016, found 411.2017.

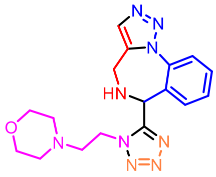

7f

4-(2-(5-(5,6-Dihydro-4*H*-benzo[*f*][1,2,3]triazolo[1,5-*a*][1,4]diazepin-6-yl)-1*H*-tetrazol-1-yl)ethyl)morpholine (7f)

According to GP and purified by column chromatography (petroleum ether/ethyl acetate 1:2) to afford a

white solid in 36% yield (26 mg). Melting point: 143–144 °C. ^{1}H NMR (400 MHz, DMSO-d_6) δ 7.99 (d, *J* = 8.0 Hz, 1H), 7.71–7.64 (m, 2H), 7.58–7.50 (m, 1H), 7.35–7.28 (m, 1H), 5.71 (d, *J* = 6.4 Hz, 1H), 4.70–4.60 (m, 1H), 4.58–4.48 (m, 1H), 4.23–4.13 (m, 1H), 4.03–3.86 (m, 2H), 3.54–3.41 (m, 4H), 2.68 (t, *J* = 6.2 Hz, 2H), 2.42–2.30 (m, 4H). ^{13}C NMR (151 MHz, DMSO-d_6) δ 155.2, 136.4, 135.8, 132.3, 130.5, 130.5, 130.4, 129.5, 123.6, 66.5, 57.3, 53.7, 53.6, 45.2, 37.5. HRMS (ESI-TOF) m/z calcd for $C_{17}H_{21}N_9NaO^+$ $[M+Na]^+$ 390.1761, found 390.1760.

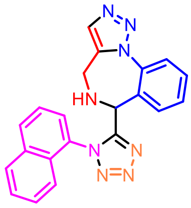

7g

6-(1-(Naphthalen-1-yl)-1*H*-tetrazol-5-yl)-5,6-dihydro-4*H*-benzo[*f*][1,2,3]triazolo[1,5-*a*][1,4]diazepine (7g)

According to GP and purified by column chromatography (petroleum ether/ethyl acetate 1:2) to afford a white solid in 85% yield (65 mg). Melting point: 160–161 °C. ^{1}H NMR (400 MHz, DMSO-d_6) δ 8.12–8.04 (m, 3H), 8.03–7.97 (m, 1H), 7.90 (d, *J* = 8.0 Hz, 1H), 7.72 (s, 1H), 7.70–7.64 (m, 2H), 7.60–7.50 (m, 2H), 7.37 (d, *J* = 4.0 Hz, 2H), 5.84 (d, *J* = 5.6 Hz, 1H), 3.97 (q, *J* = 5.4 Hz, 1H), 3.91–3.77 (m, 2H). ^{13}C NMR (101 MHz, DMSO-d_6) δ 156.3, 136.2, 136.0, 133.4, 132.7, 132.3, 132.0, 131.1, 130.4, 130.1, 129.8, 129.3, 128.8, 128.3, 128.2, 127.9, 124.7, 123.6, 123.1, 54.1, 37.3. HRMS (ESI-TOF) m/z calcd for $C_{21}H_{16}N_8Na^+$ $[M+Na]^+$ 403.1390, found 403.1390.

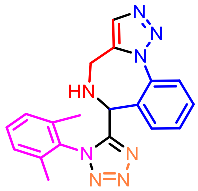

7h

6-(1-(2,6-Dimethylphenyl)-1*H*-tetrazol-5-yl)-5,6-dihydro-4*H*-benzo[*f*][1,2,3]triazolo[1,5-*a*][1,4]diazepine (7h)

According to GP and purified by column chromatography (petroleum ether/ethyl acetate 1:2) to afford a white solid in 83% yield (59 mg). Melting point: 159–161 °C. ^{1}H NMR (400 MHz, DMSO-d_6) δ 7.86 (d, *J* = 8.0 Hz, 1H), 7.76 (s, 1H), 7.62 (td, *J* = 7.6, 1.2 Hz, 1H), 7.44–7.28 (m, 3H), 7.20–7.09 (m, 2H), 5.32 (d, *J* = 6.4 Hz, 1H), 4.11 (dd, *J* = 14.4, 4.0 Hz, 1H), 3.93 (q, *J* = 5.4 Hz, 1H), 3.74 (dd, *J* = 14.4, 5.6 Hz, 1H), 1.91 (s, 3H), 1.48 (s, 3H). ^{13}C NMR (101 MHz, DMSO-d_6) δ 157.0, 136.2, 136.0, 135.8, 135.5, 132.4, 132.0, 131.3, 131.2, 130.7, 129.5, 129.1, 128.9, 128.9, 123.8, 54.3, 37.4, 17.4, 16.9. HRMS (ESI-TOF) m/z calcd for $C_{19}H_{18}N_8Na^+$ $[M+Na]^+$ 381.1547, found 381.1547.

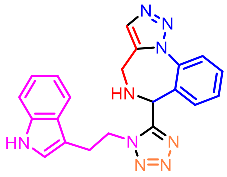

7i

6-(1-(2-(1*H*-Indol-3-yl)ethyl)-1*H*-tetrazol-5-yl)-5,6-dihydro-4*H*-benzo[*f*][1,2,3]triazolo[1,5-*a*][1,4]diazepine (7i)

According to GP and purified by column chromatography (petroleum ether/ethyl acetate 1:2) to afford a yellow solid in 89% yield (71 mg). Melting point: 152–153 °C. ^{1}H NMR (400 MHz, $CDCl_3$) δ 8.52 (s, 1H), 7.81 (d, *J* = 8.0 Hz, 1H), 7.62 (s, 1H), 7.50 (t, *J* = 7.8 Hz, 1H), 7.34 (d, *J* = 8.0 Hz, 1H), 7.30–7.22 (m, 2H), 7.17 (t, *J* = 7.6 Hz, 1H), 6.99 (t, *J* = 7.4 Hz, 1H), 6.83 (d, *J* = 8.0 Hz, 1H), 6.63 (d, *J* = 2.0 Hz, 1H), 6.55 (d, *J* = 7.6 Hz, 1H), 4.70–4.55 (m, 1H), 4.06 (s, 1H), 4.02–3.90 (m, 1H), 3.83 (d, *J* = 14.8 Hz, 1H), 3.55 (d, *J* = 14.4 Hz, 1H), 3.30–3.18 (m, 1H), 3.17–3.10 (m, 1H). ^{13}C NMR (101 MHz, $CDCl_3$) δ 154.4, 135.8, 134.6, 131.9, 130.4, 129.7, 128.2, 128.1, 126.4, 123.6, 122.8, 122.5, 120.2, 117.3, 111.5, 110.4, 51.3, 49.6, 37.5, 25.8. HRMS (ESI-TOF) m/z calcd for $C_{21}H_{19}N_9Na^+$ $[M+Na]^+$ 420.1656, found 420.1651.

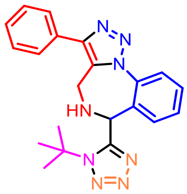

7j

6-(1-(*tert*-Butyl)-1*H*-tetrazol-5-yl)-3-phenyl-5,6-dihydro-4*H*-benzo[*f*][1,2,3]triazolo[1,5-*a*][1,4]diazepine (7j)

According to GP and purified by column chromatography (petroleum ether/ethyl acetate 1:2) to afford a yellow solid in 85% yield (66 mg). Melting point: 162–163 °C. ^{1}H NMR (400 MHz, $CDCl_3$) δ 7.96 (d, *J* = 7.6 Hz, 1H), 7.85–7.78 (m, 2H), 7.60 (t, *J* = 7.8 Hz, 1H), 7.49–7.35 (m, 4H), 6.69 (d, *J* = 7.6 Hz, 1H), 5.36 (s, 1H), 4.50 (dd, *J* = 13.2, 1.2 Hz, 1H), 3.89 (d, *J* = 13.2 Hz, 1H), 1.40 (s, 9H). ^{13}C NMR (101 MHz, $CDCl_3$) δ 152.7, 145.4, 135.6, 130.5, 130.2, 130.1, 129.9, 129.1, 128.6, 128.0, 127.4, 123.8, 62.2, 53.0, 38.5, 29.8. HRMS (ESI-TOF) m/z calcd for $C_{21}H_{22}N_8Na^+$ $[M+Na]^+$ 409.1860, found 409.1862.

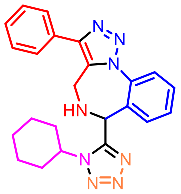

7k

6-(1-Cyclohexyl-1*H*-tetrazol-5-yl)-3-phenyl-5,6-dihydro-4*H*-benzo[*f*][1,2,3]triazolo[1,5-*a*][1,4]diazepine (7k)

According to GP and purified by column chromatography (petroleum ether/ethyl acetate 1:2) to afford a white solid in 84% yield (69 mg). Melting point: 157–158 °C. ^{1}H NMR (400 MHz, DMSO-d_6) δ 8.00 (dd, *J* = 8.0, 1.2 Hz, 1H), 7.74–7.68 (m, 3H), 7.60 (td, *J* = 7.6, 1.2 Hz, 1H), 7.50 (t, *J* = 7.6 Hz, 2H), 7.46–7.38

(m, 2H), 5.91 (d, J = 6.4 Hz, 1H), 4.70–4.58 (m, 1H), 4.35 (q, J = 5.3 Hz, 1H), 4.12 (dd, J = 14.4, 4.8 Hz, 1H), 3.98 (dd, J = 14.8, 5.2 Hz, 1H), 1.96–1.85 (m, 1H), 1.80–1.65 (m, 4H), 1.64–1.52 (m, 2H), 1.35–1.15 (m, 3H). ^{13}C NMR (101 MHz, DMSO-d_6) δ 154.1, 142.9, 135.9, 131.7, 130.5, 130.4, 130.1, 129.7, 129.5, 128.9, 128.1, 126.8, 123.4, 57.2, 53.3, 37.7, 32.5, 32.0, 24.7, 24.5. HRMS (ESI-TOF) m/z calcd for $C_{23}H_{24}N_8Na^+$ [M+Na]$^+$ 435.2016, found 435.2020.

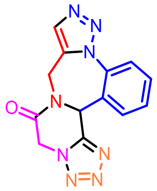

8a

8*H*,16b*H*-Benzo[*f*]tetrazolo[5',1':3,4]pyrazino[1,2-*d*][1,2,3]triazolo[1,5-*a*][1,4]diazepin-6(5*H*)-one (8a)

Gram-scale synthesis of **8a**: A solution of 2-azidobenzaldehyde (**1a**, 10 mmol, 1 equiv) and propargylamine (**2a**, 10 mmol, 1 equiv) in MeOH (15 mL) was heated at 40 °C for 40 min, then 2-isocyanoacetate (**9**, 10 mmol, 1 equiv) and $TMSN_3$ (**4**, 10 mmol, 1 equiv) was added followed by heating at 40 °C for 12 h. After evaporating MeOH solvent, the residue was redissolved in MeCN (15 mL) and heated at 130 °C for 2 h in a sealed vial. The completion of the reaction was monitored by TLC and a white solid precipitated upon cooling. After concentration in vacuo, the crude product was purified by chromatography on silica gel with 1:2 petroleum ether/ethyl acetate to afford 2.67 g (91%) of product **8a**.

According to GP and purified by column chromatography (petroleum ether/ethyl acetate 1:2) to afford a white solid in 92% yield (54 mg). Melting point: 172–173 °C. ^{1}H NMR (400 MHz, DMSO-d_6) δ 8.14 (s, 1H), 8.00 (d, J = 8.0 Hz, 1H), 7.76 (t, J = 7.6 Hz, 1H), 7.57 (t, J = 7.6 Hz, 1H), 6.98 (d, J = 8.0 Hz, 1H), 6.22 (s, 1H), 5.49 (d, J = 16.0 Hz, 1H), 5.45–5.35 (m, 2H), 4.17 (d, J = 15.6 Hz, 1H). ^{13}C NMR (101 MHz, DMSO-d_6) δ 160.3, 147.8, 135.1, 134.4, 131.4, 131.2, 129.6, 128.6, 127.7, 123.1, 51.6, 47.9, 37.8. HRMS (ESI-TOF) m/z calcd for $C_{13}H_{10}N_8NaO^+$ [M+Na]$^+$ 317.0870, found 317.0877.

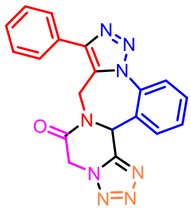

8b

9-Phenyl-8*H*,16b*H*-benzo[*f*]tetrazolo[5',1':3,4]pyrazino[1,2-*d*][1,2,3]triazolo[1,5-*a*][1,4]diazepin-6(5*H*)-one (8b)

According to GP and purified by column chromatography (petroleum ether/ethyl acetate 1:2) to afford a faint yellow solid in 78% yield (58 mg). Melting point: 166–168 °C. ^{1}H NMR (400 MHz, DMSO-d_6) δ 8.07 (dd, J = 8.0, 1.6 Hz, 1H), 7.83–7.75 (m, 3H), 7.66–7.61 (m, 1H), 7.59–7.54 (m, 2H), 7.52–7.45 (m, 1H), 7.15 (dd, J = 7.6, 1.2 Hz, 1H), 6.28 (s, 1H), 5.59 (d, J = 15.6 Hz, 1H), 5.45 (dd, J = 17.2, 2.0 Hz, 1H), 5.37 (dd, J = 17.2, 2.0 Hz, 1H), 4.18 (d, J = 16.0 Hz, 1H). ^{13}C NMR (101 MHz, DMSO-d_6) δ 167.3, 160.4, 147.8,

145.4, 135.1, 131.6, 131.3, 129.8, 129.8, 129.0, 129.0, 128.7, 128.7, 127.7, 127.6, 127.4, 123.2, 51.9, 48.0, 37.5. HRMS (ESI-TOF) m/z calcd for $C_{19}H_{14}N_8NaO^+$ $[M+Na]^+$ 393.1183, found 393.1182.

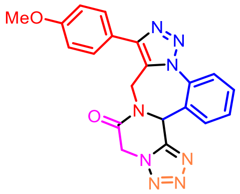

8c

9-(4-Methoxyphenyl)-8*H*,16b*H*-benzo[*f*]tetrazolo[5',1':3,4]pyrazino[1,2-*d*][1,2,3]triazolo[1,5-*a*][1,4]diazepin-6(5*H*)-one (8c)

According to GP and purified by column chromatography (petroleum ether/ethyl acetate 1:2) to afford a white solid in 81% yield (65 mg). Melting point: 160–162 °C. ^{1}H NMR (300 MHz, DMSO-d_6) δ 8.05 (d, J = 7.8 Hz, 1H), 7.80 (t, J = 7.8 Hz, 1H), 7.69 (d, J = 8.1 Hz, 2H), 7.62 (t, J = 7.7 Hz, 1H), 7.14 (t, J = 7.8 Hz, 3H), 6.27 (s, 1H), 5.58 (d, J = 15.6 Hz, 1H), 5.46 (d, J = 17.4 Hz, 1H), 5.36 (d, J = 17.4 Hz, 1H), 4.16 (d, J = 15.6 Hz, 1H), 3.83 (s, 3H). ^{13}C NMR (75 MHz, DMSO-d_6) δ 160.8, 160.1, 148.3, 145.8, 135.7, 131.8, 130.2, 129.4, 127.8, 127.4, 123.6, 122.6, 115.0, 55.7, 52.4, 48.5, 38.0. HRMS (ESI-TOF) m/z calcd for $C_{20}H_{16}N_8NaO_2^+$ $[M+Na]^+$ 423.1288, found 423.1284.

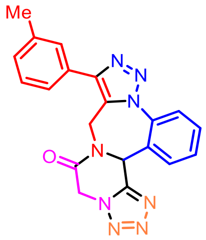

8d

9-(*m*-Tolyl)-8*H*,16b*H*-benzo[*f*]tetrazolo[5',1':3,4]pyrazino[1,2-*d*][1,2,3]triazolo[1,5-*a*][1,4]diazepin-6(5*H*)-one (8d)

According to GP and purified by column chromatography (petroleum ether/ethyl acetate 1:2) to afford a white solid in 80% yield (62 mg). Melting point: 165–167 °C. ^{1}H NMR (400 MHz, DMSO-d_6) δ 8.06 (d, J = 8.0 Hz, 1H), 7.81 (t, J = 7.4 Hz, 1H), 7.67–7.58 (m, 2H), 7.54 (d, J = 7.6 Hz, 1H), 7.44 (t, J = 7.6 Hz, 1H), 7.30 (d, J = 7.6 Hz, 1H), 7.16 (d, J = 7.6 Hz, 1H), 6.27 (s, 1H), 5.60 (d, J = 15.7 Hz, 1H), 5.45 (dd, J = 17.6, 2.0 Hz, 1H), 5.37 (dd, J = 17.2, 2.0 Hz, 1H), 4.17 (d, J = 15.6 Hz, 1H), 2.40 (s, 3H). ^{13}C NMR (101 MHz, DMSO-d_6) δ 160.4, 147.8, 145.5, 138.3, 135.2, 131.3, 129.8, 129.7, 129.3, 129.0, 128.9, 128.1, 127.6, 127.3, 124.7, 123.2, 51.9, 48.0, 37.5, 21.0. HRMS (ESI-TOF) m/z calcd for $C_{20}H_{16}N_8NaO^+$ $[M+Na]^+$ 407.1339, found 407.1332.

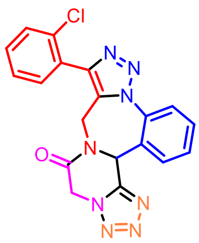

8e

9-(2-Chlorophenyl)-8*H*,16b*H*-benzo[*f*]tetrazolo[5',1':3,4]pyrazino[1,2-*d*][1,2,3]triazolo[1,5-*a*][1,4]diazepin-6(5*H*)-one (8e)

According to GP and purified by column chromatography (petroleum ether/ethyl acetate 1:2) to afford a faint yellow solid in 77% yield (62 mg). Melting point: 177–178 °C. ^{1}H NMR (400 MHz, DMSO-d_6) δ 8.09 (dd, J = 8.0, 1.2 Hz, 1H), 7.81 (td, J = 7.6, 1.2 Hz, 1H), 7.70–7.59 (m, 3H), 7.58 – 7.50 (m, 2H), 7.04 (dd, J = 7.6, 1.2 Hz, 1H), 6.35 (s, 1H), 5.43 (dd, J = 17.2, 1.6 Hz, 1H), 5.36 (dd, J = 17.2, 2.0 Hz, 1H), 5.16 (d, J = 15.6 Hz, 1H), 4.20 (d, J = 15.6 Hz, 1H). ^{13}C NMR (101 MHz, DMSO-d_6) δ 160.2, 147.7, 143.6, 134.9, 132.6, 132.4, 131.3, 131.0, 130.0, 129.8, 129.2, 128.8, 128.3, 127.7, 127.5, 123.2, 51.7, 47.9, 38.0. HRMS (ESI-TOF) m/z calcd for $C_{19}H_{13}ClN_8NaO^+$ [M+Na]$^+$ 427.0793, found 427.0799.

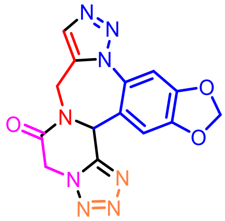

8f

4b*H*,12*H*-[1,3]Dioxolo[4',5':4,5]benzo[1,2-*f*]tetrazolo[5',1':3,4]pyrazino[1,2-*d*][1,2,3]triazolo[1,5-*a*][1,4]diazepin-10(9*H*)-one (8f)

According to GP and purified by column chromatography (petroleum ether/ethyl acetate 1:2) to afford a faint yellow solid in 77% yield (52 mg). Melting point: 159–162 °C. ^{1}H NMR (400 MHz, DMSO-d_6) δ 8.10 (s, 1H), 7.54 (s, 1H), 6.52 (s, 1H), 6.21 (d, J = 6.8 Hz, 2H), 6.00 (s, 1H), 5.48 (d, J = 15.2 Hz, 1H), 5.36 (s, 2H), 4.07 (d, J = 15.6 Hz, 1H). ^{13}C NMR (101 MHz, DMSO-d_6) δ 160.1, 149.1, 147.9, 147.8, 134.2, 131.4, 129.6, 121.5, 107.9, 104.0, 102.9, 51.4, 47.8, 37.6. HRMS (ESI-TOF) m/z calcd for $C_{14}H_{10}N_8NaO_3^+$ [M+Na]$^+$ 361.0768, found 361.0769.

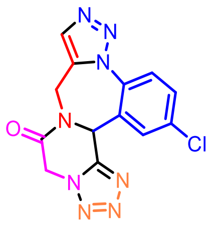

8g

15-Chloro-8*H*,16b*H*-benzo[*f*]tetrazolo[5',1':3,4]pyrazino[1,2-*d*][1,2,3]triazolo[1,5-*a*][1,4]diazepin-6(5*H*)-one (8g)

According to GP and purified by column chromatography (petroleum ether/ethyl acetate 1:2) to afford a white solid in 79% yield (52 mg). Melting point: 169–171 °C. ^{1}H NMR (400 MHz, DMSO-d_6) δ 8.14 (s,

1H), 8.02 (d, J = 8.4 Hz, 1H), 7.84 (dd, J = 8.4, 2.4 Hz, 1H), 7.12 (d, J = 2.4 Hz, 1H), 6.25 (s, 1H), 5.49 (d, J = 15.6 Hz, 1H), 5.37 (s, 2H), 4.19 (d, J = 15.6 Hz, 1H). ^{13}C NMR (101 MHz, DMSO-d_6) δ 160.7, 147.9, 135.0, 134.6, 134.1, 132.0, 131.7, 129.8, 129.4, 125.4, 51.9, 48.4, 38.1. HRMS (ESI-TOF) m/z calcd for $C_{13}H_9ClN_8NaO^+$ [M+Na]$^+$ 351.0480, found 351.0479.

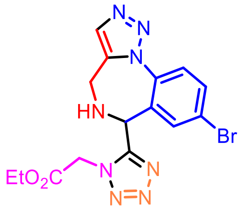

8h'

Ethyl 2-(5-(8-bromo-5,6-dihydro-4*H*-benzo[*f*][1,2,3]triazolo[1,5-*a*][1,4]diazepin-6-yl)-1*H*-tetrazol-1-yl)acetate (8h')

According to GP and purified by column chromatography (petroleum ether/ethyl acetate 1:2) to afford a white solid in 59% yield (49 mg). Melting point: 138–140 °C. ^{1}H NMR (400 MHz, DMSO-d_6) δ 7.06 (d, J = 8.4 Hz, 1H), 7.00 (dd, J = 8.4, 2.0 Hz, 1H), 6.90 (d, J = 2.0 Hz, 1H), 6.74 (s, 1H), 4.90 (d, J = 6.8 Hz, 1H), 4.68 (d, J = 17.6 Hz, 1H), 4.60 (d, J = 17.6 Hz, 1H), 3.38–3.29 (m, 3H), 3.03 (dd, J = 14.4, 2.8 Hz, 1H), 2.92 (dd, J = 14.4, 5.2 Hz, 1H), 0.37 (t, J = 7.2 Hz, 3H). ^{13}C NMR (101 MHz, DMSO-d_6) δ 166.2, 155.1, 135.1, 134.6, 133.3, 132.8, 132.5, 132.3, 125.1, 121.4, 61.7, 53.5, 49.2, 36.6, 14.0. HRMS (ESI-TOF) m/z calcd for $C_{15}H_{15}BrN_8NaO_2^+$ [M+Na]$^+$ 441.0394, found 441.0394.

6. NMR spectra of compounds 7 and 8

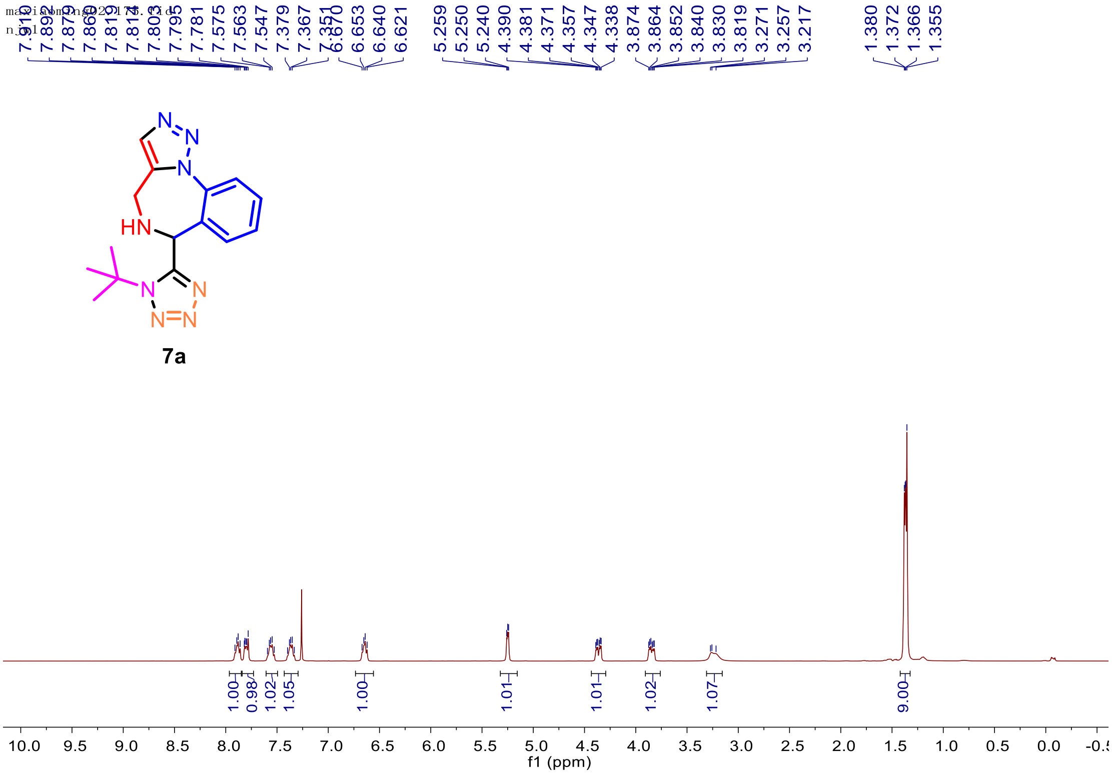

^{1}H NMR spectrum of compound 7a (400 MHz, $CDCl_3$)

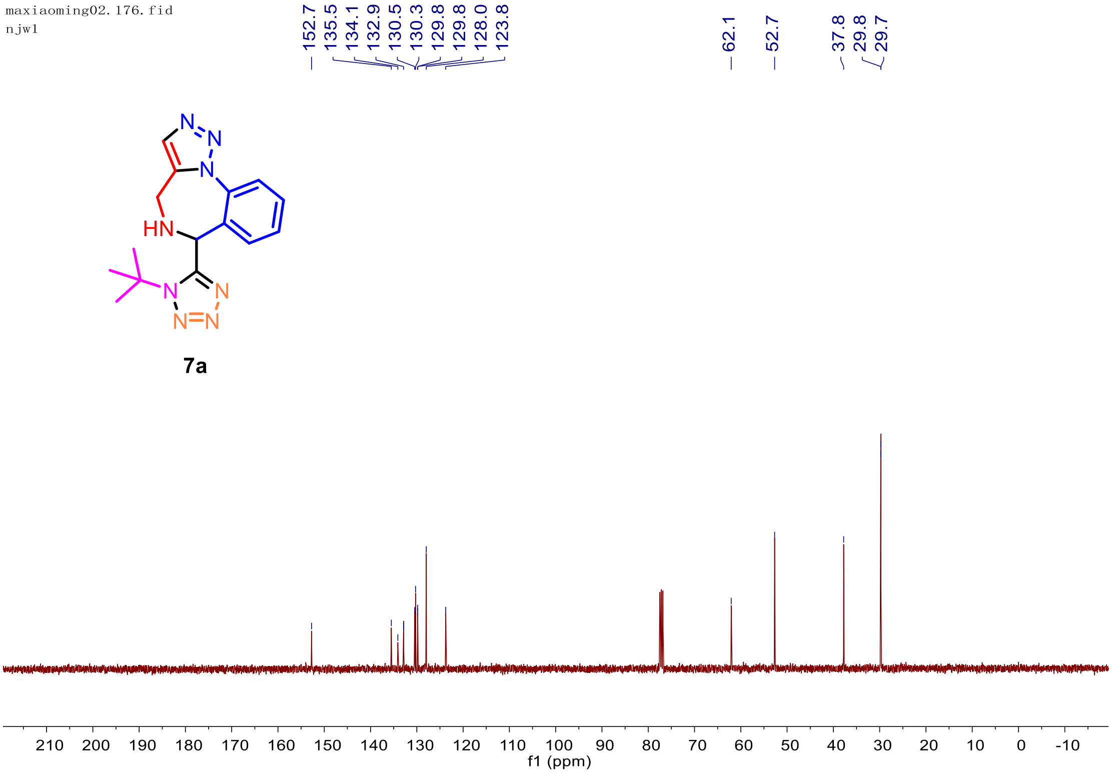

^{13}C NMR spectrum of compound 7a (101 MHz, $CDCl_3$)

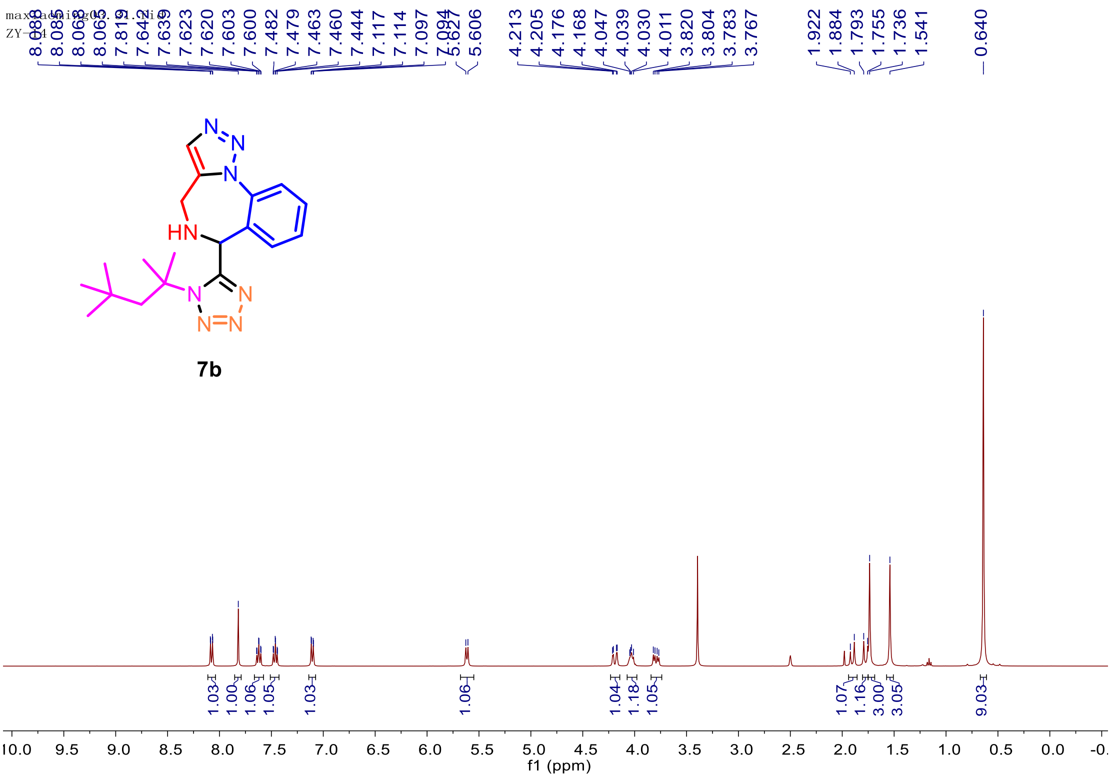

^{1}H NMR spectrum of compound 7b (400 MHz, DMSO-*d*6)

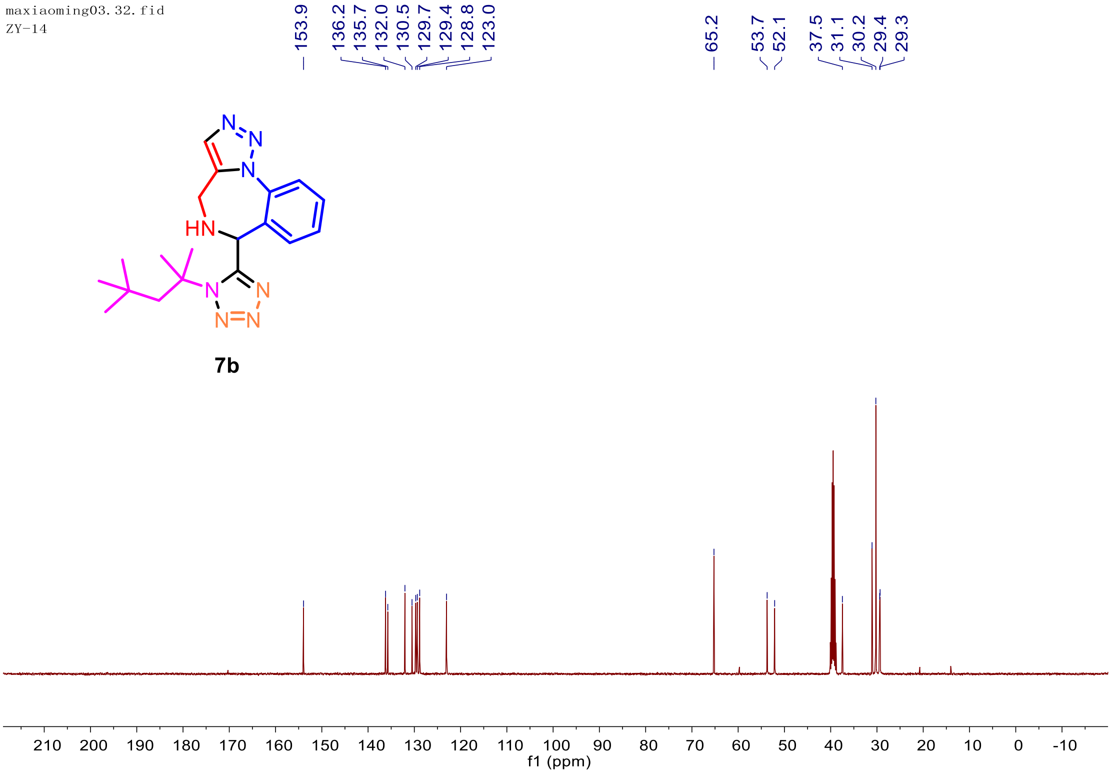

^{13}C NMR spectrum of compound 7b (101 MHz, DMSO-*d*6)

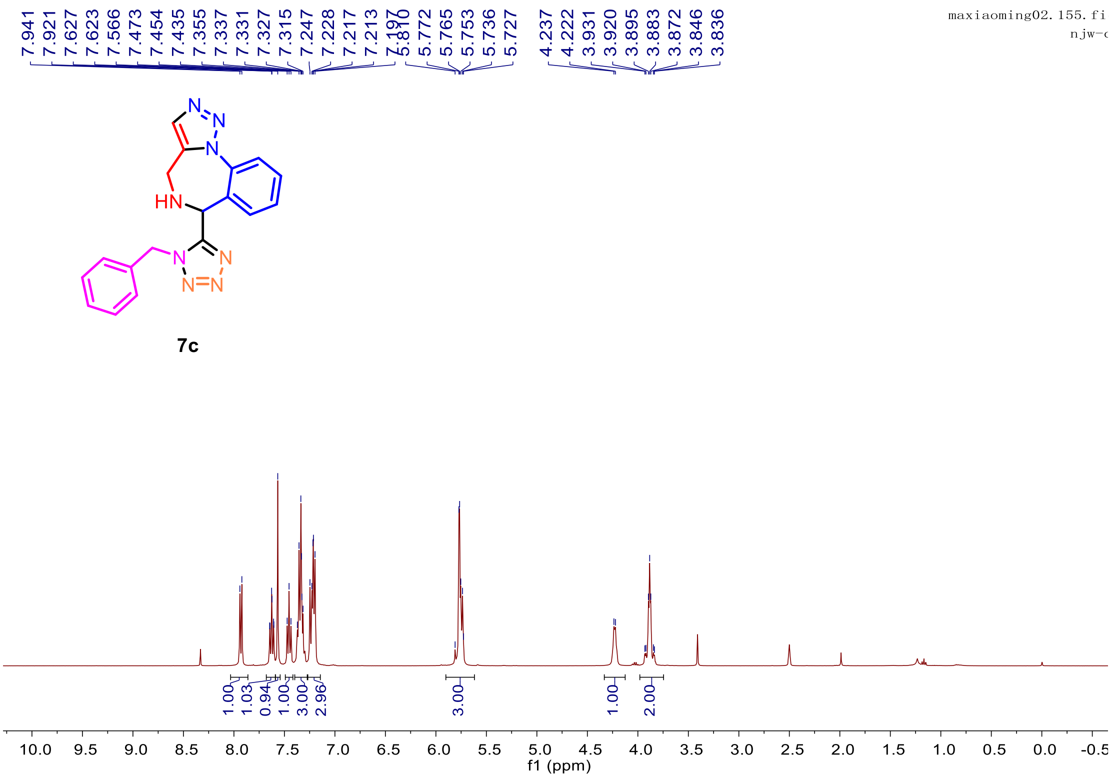

^{1}H NMR spectrum of compound 7c (400 MHz, DMSO-*d*6)

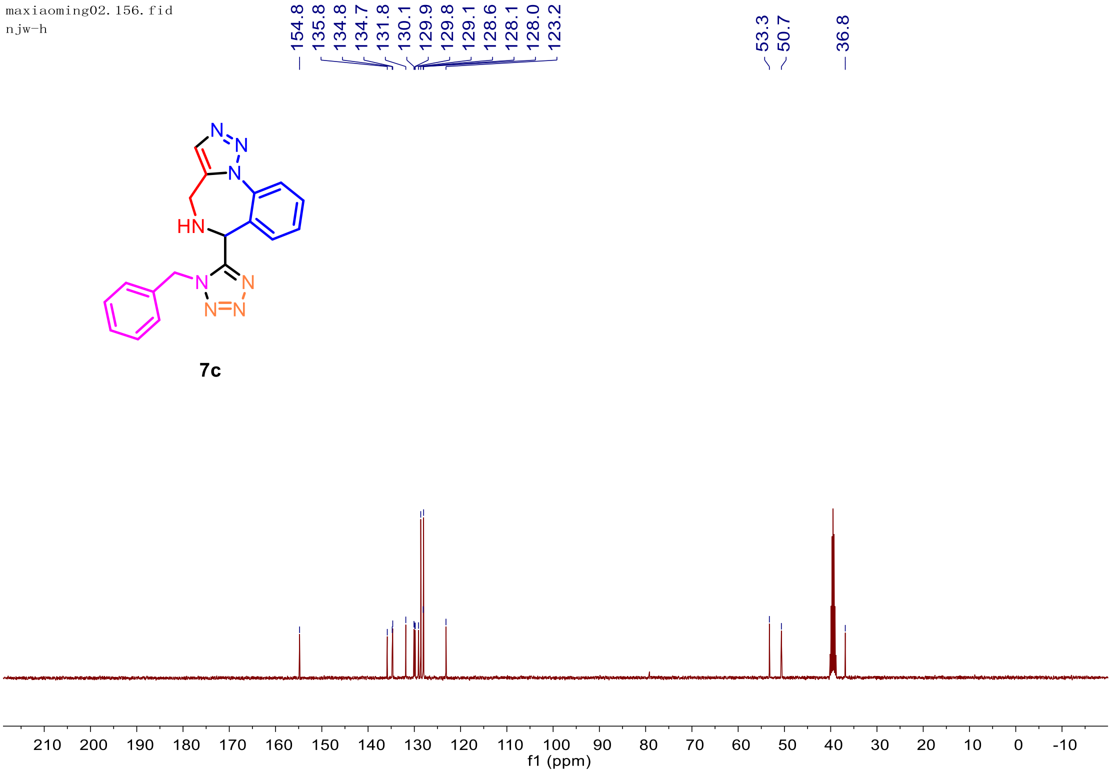

^{13}C NMR spectrum of compound 7c (101 MHz, DMSO-*d*6)

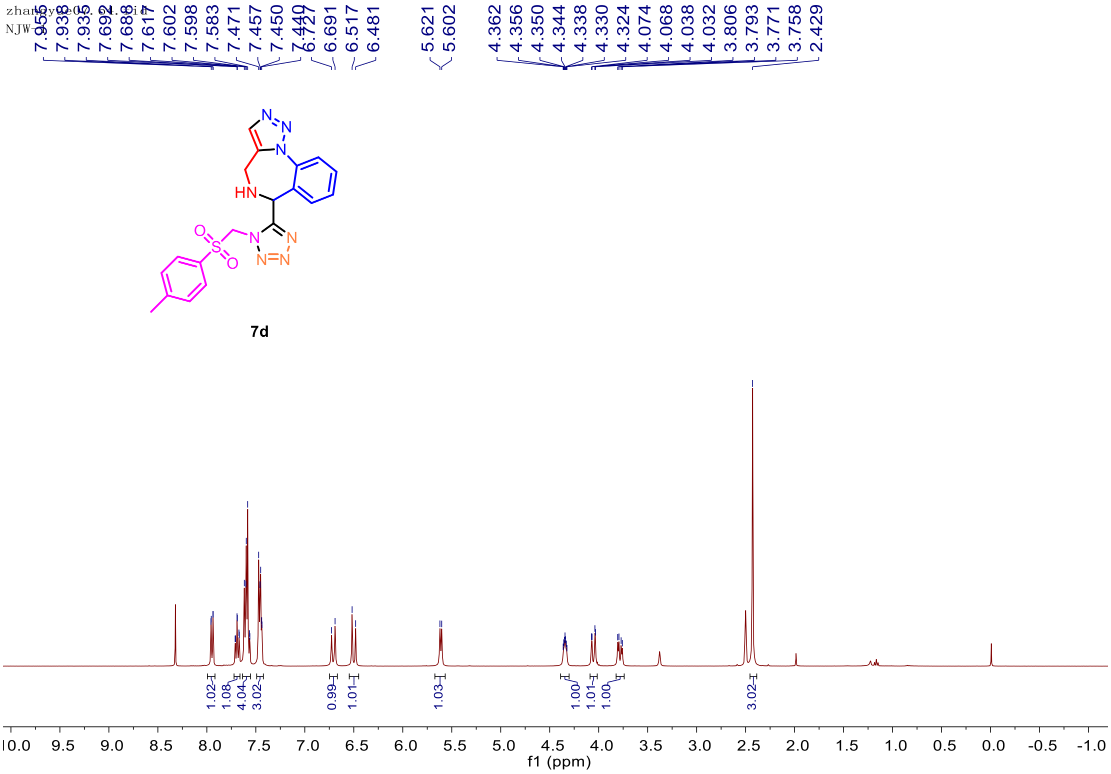

^{1}H NMR spectrum of compound 7d (400 MHz, DMSO-d_6)

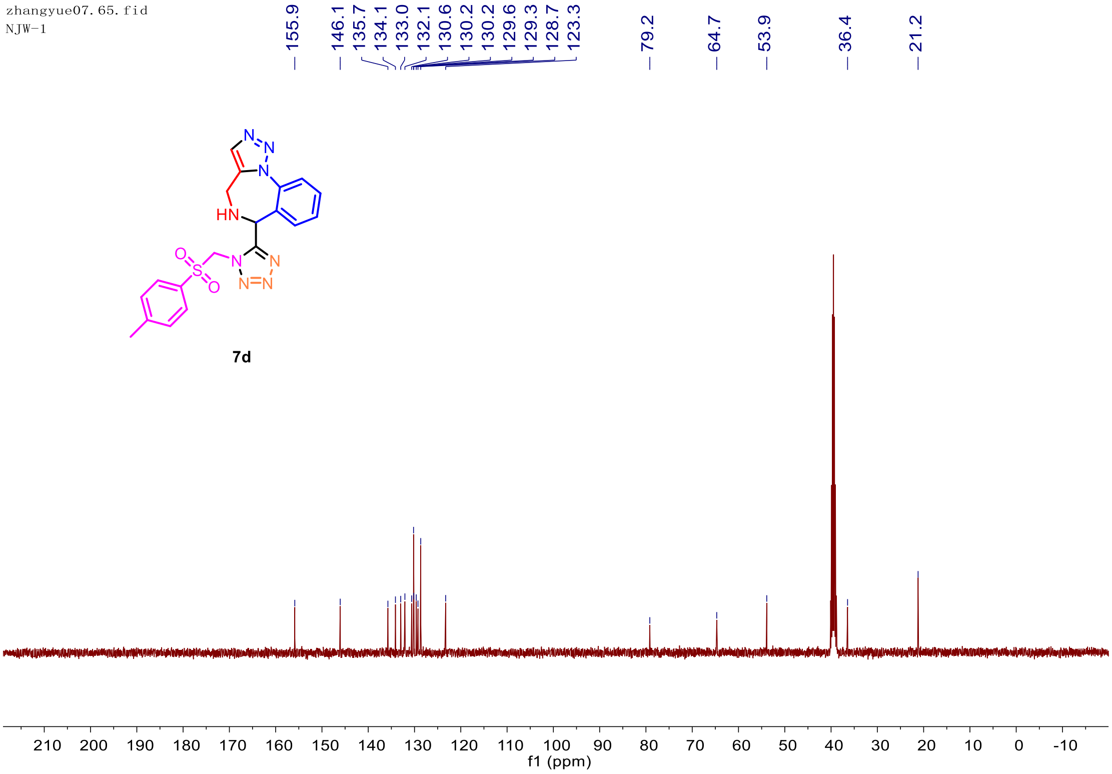

^{13}C NMR spectrum of compound 7d (101 MHz, DMSO-d_6)

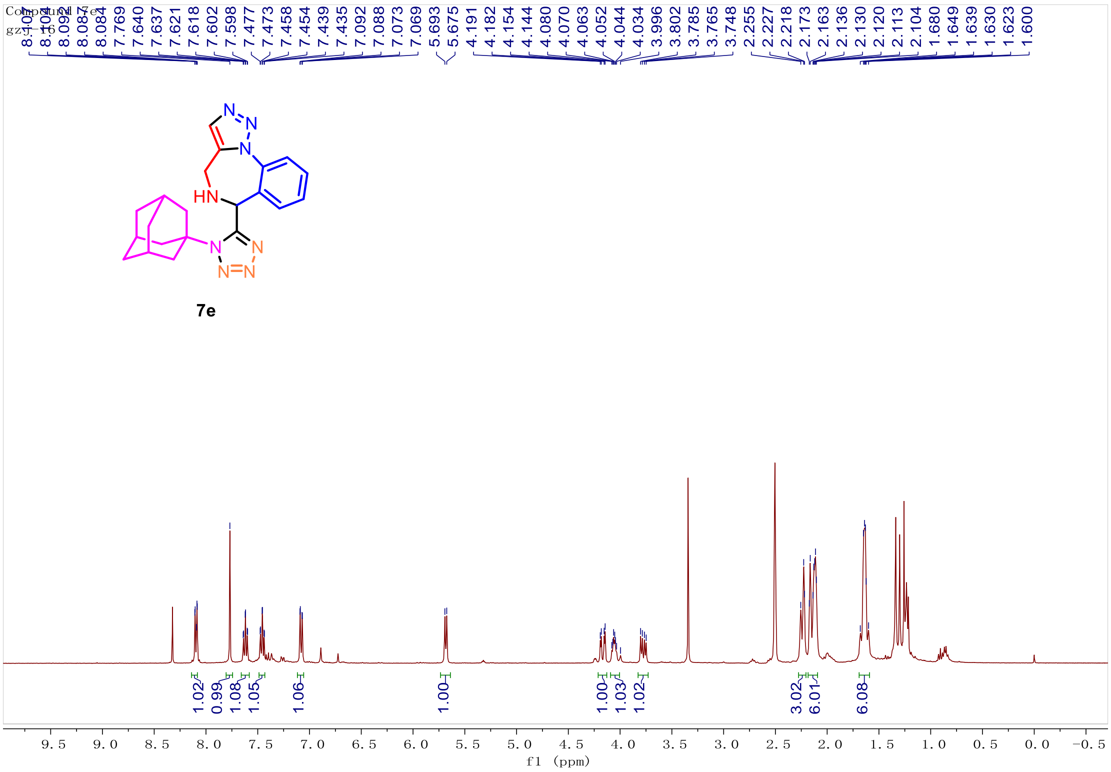

^{1}H NMR spectrum of compound 7e (400 MHz, DMSO-d_6)

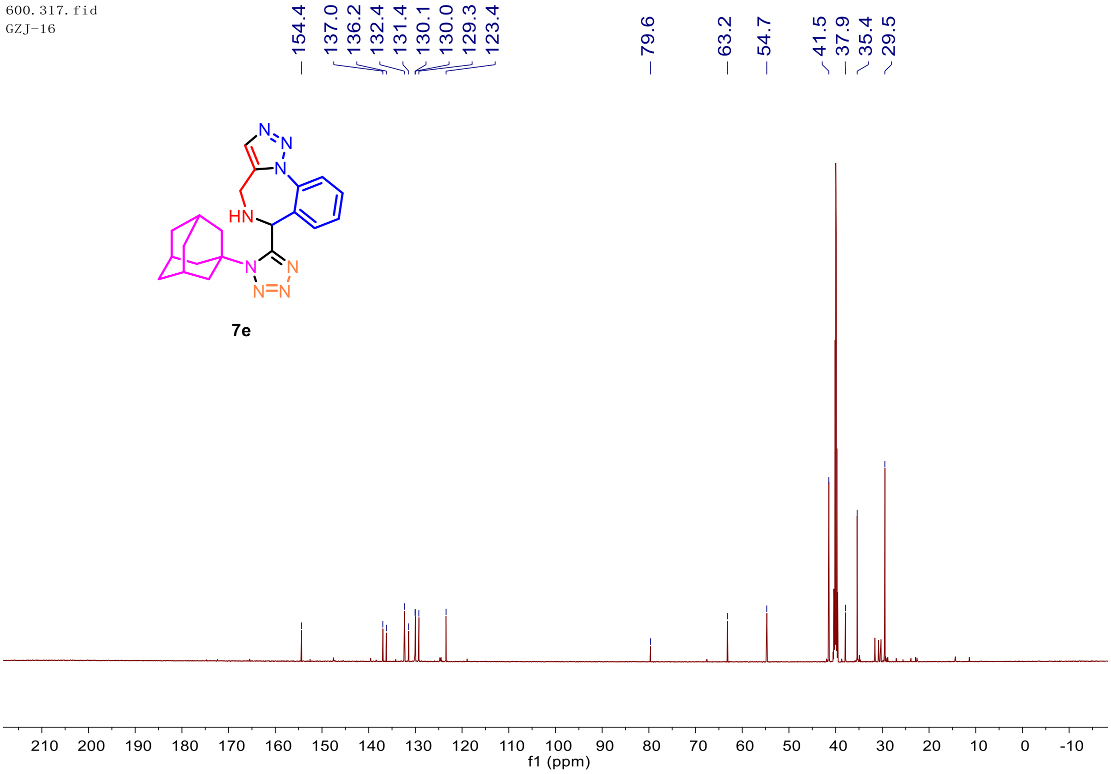

^{13}C NMR spectrum of compound 7e (151 MHz, DMSO-d_6)

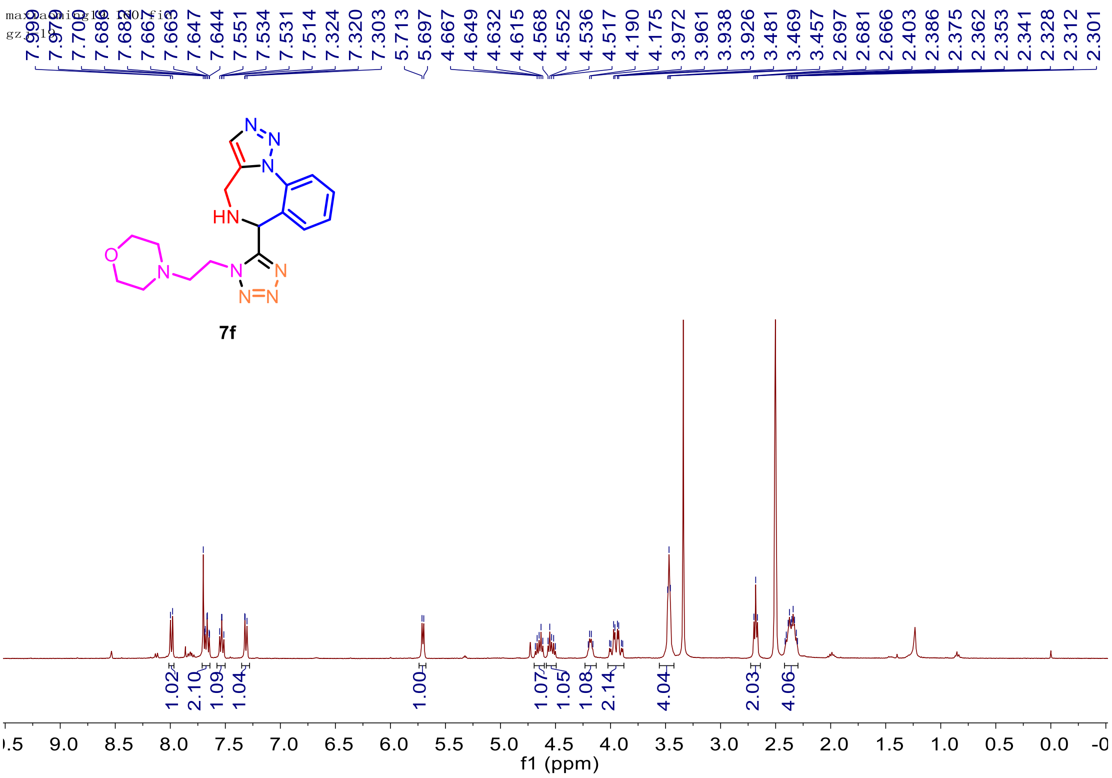

^1H NMR spectrum of compound 7f (400 MHz, DMSO-d_6)

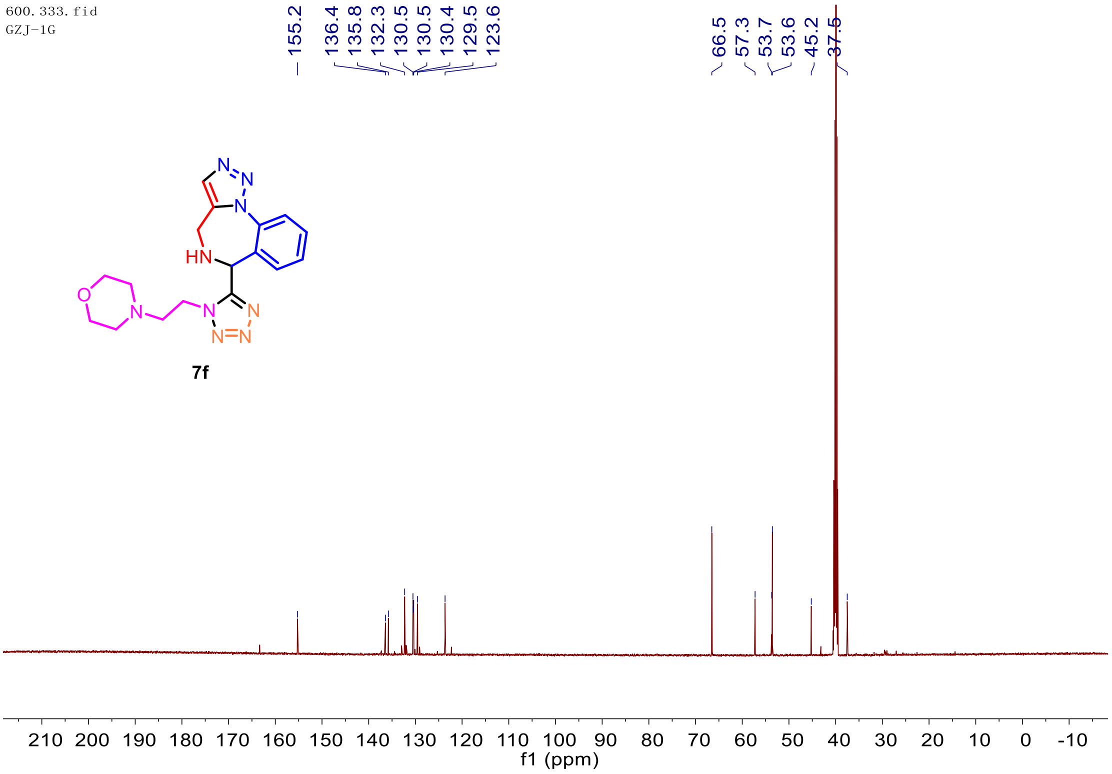

^{13}C NMR spectrum of compound 7f (151 MHz, DMSO-d_6)

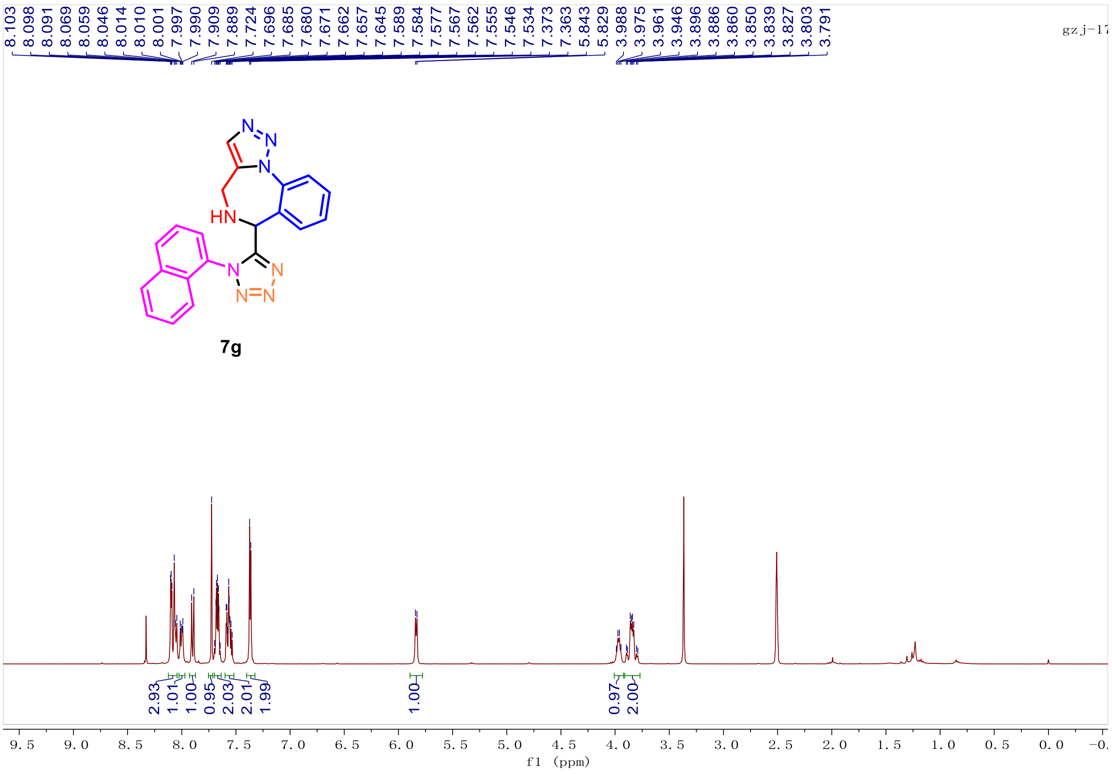

^{1}H NMR spectrum of compound 7g (400 MHz, DMSO-d_6)

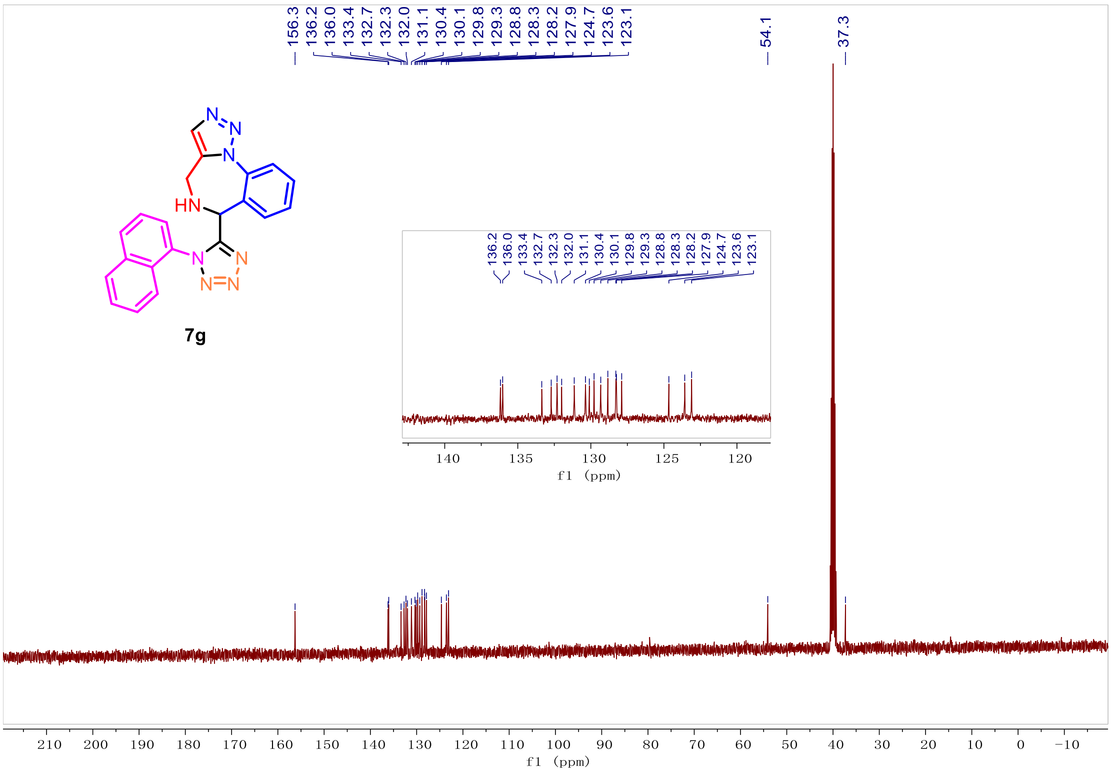

^{13}C NMR spectrum of compound 7g (101 MHz, DMSO-d_6)

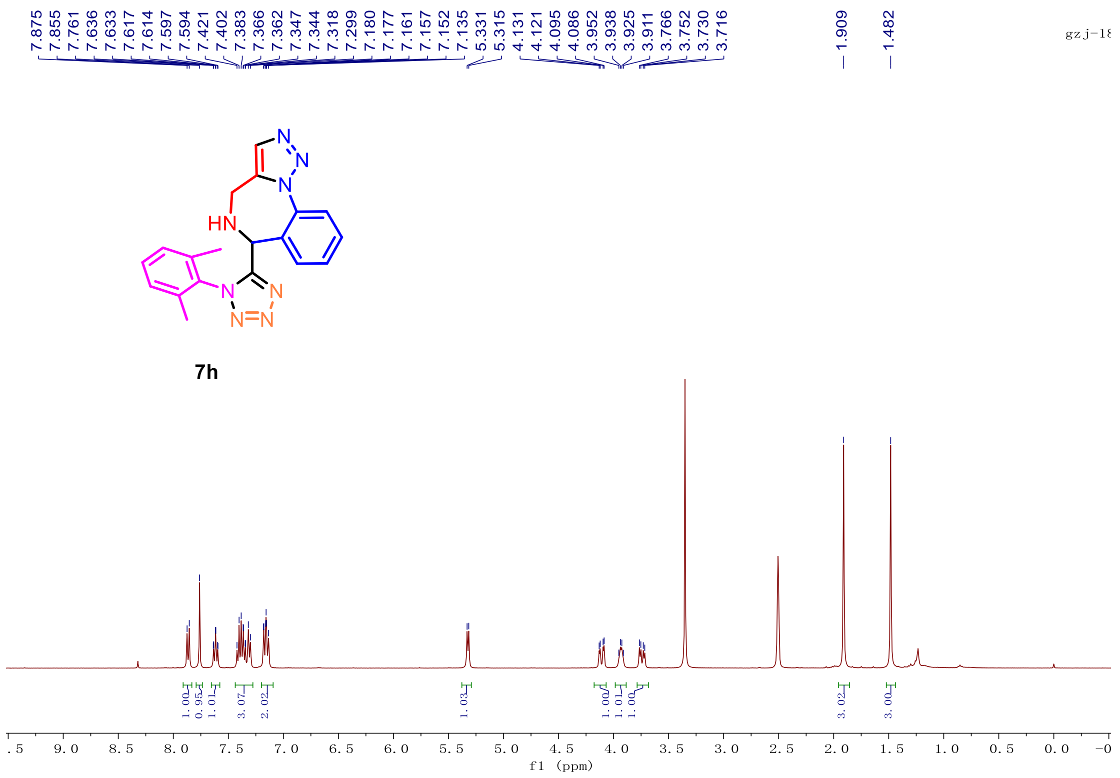

^{1}H NMR spectrum of compound 7h (400 MHz, DMSO-d_6)

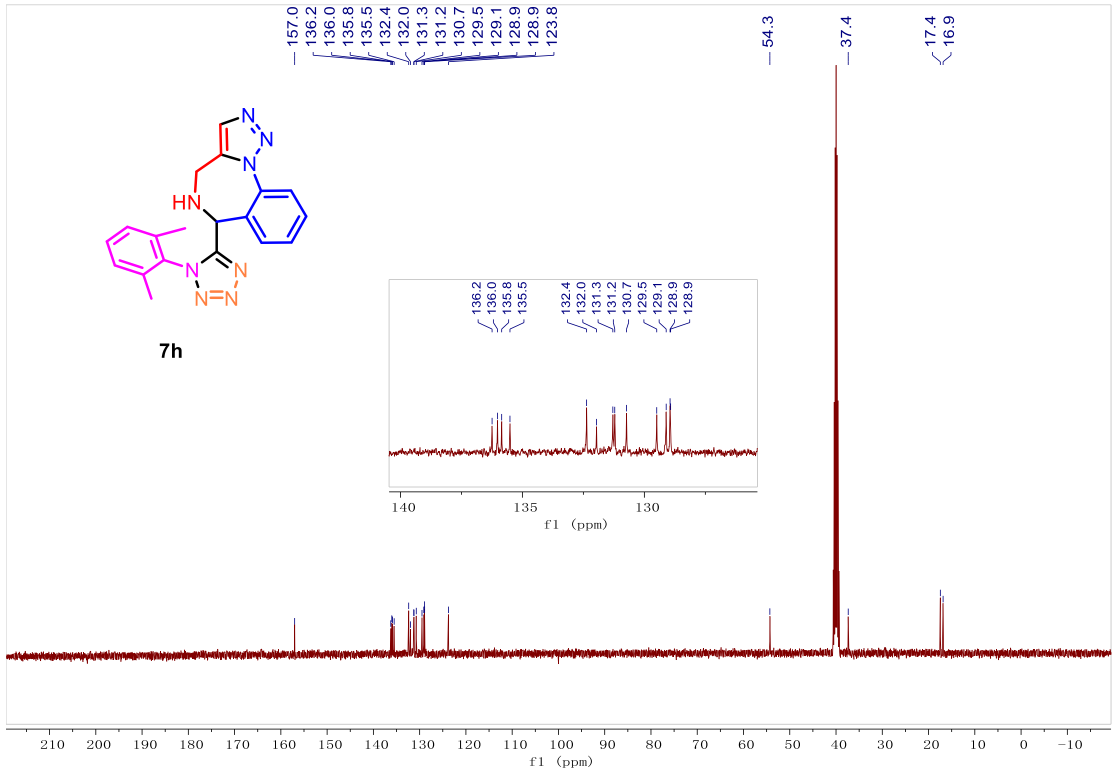

^{13}C NMR spectrum of compound 7h (101 MHz, DMSO-d_6)

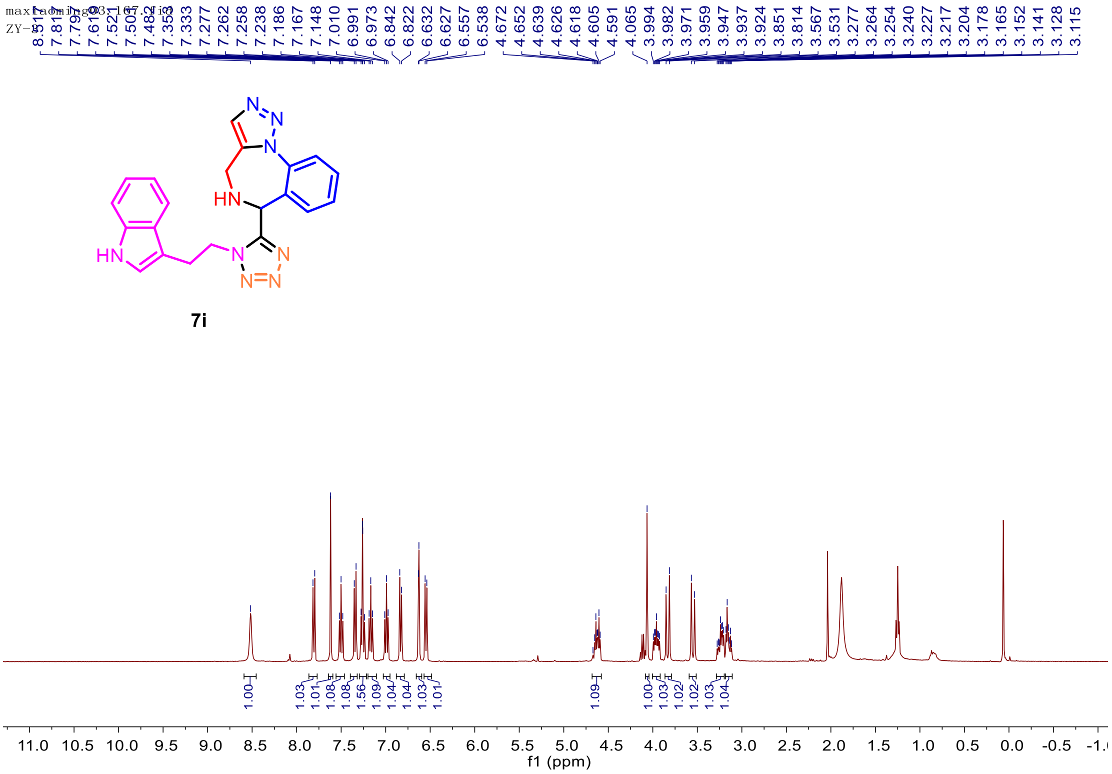

^{1}H NMR spectrum of compound 7i (400 MHz, CDCl$_3$)

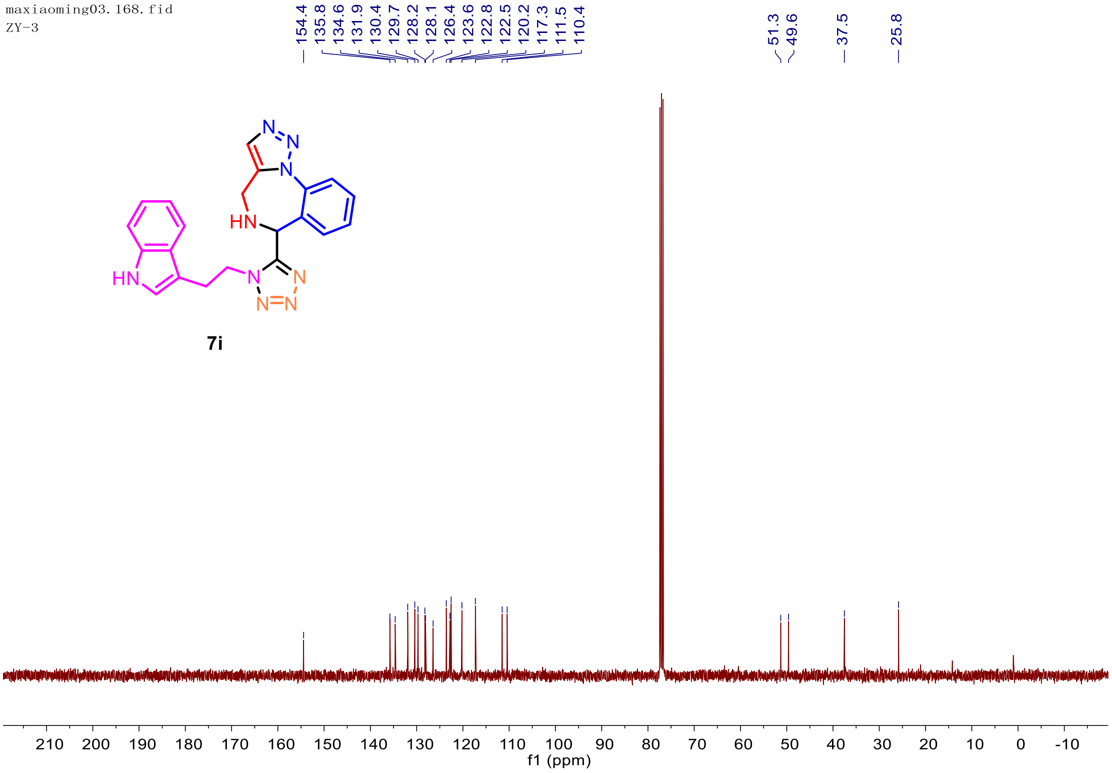

^{13}C NMR spectrum of compound 7i (101 MHz, CDCl$_3$)

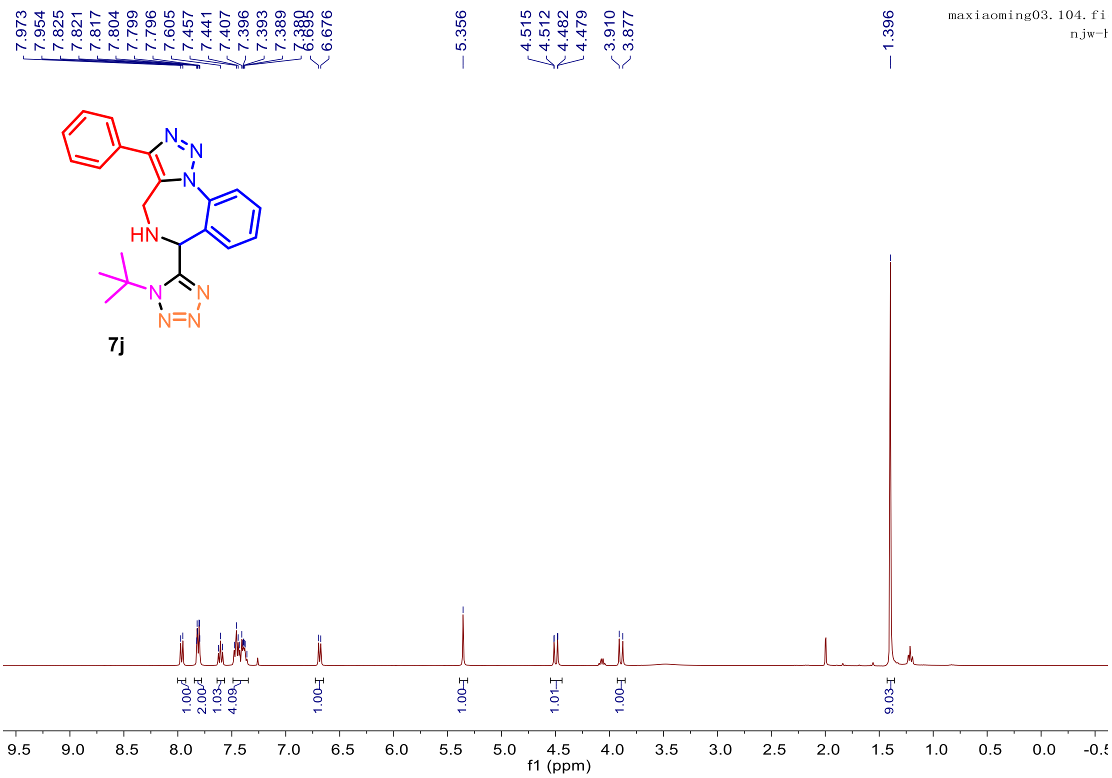

^{1}H NMR spectrum of compound 7j (400 MHz, $CDCl_3$)

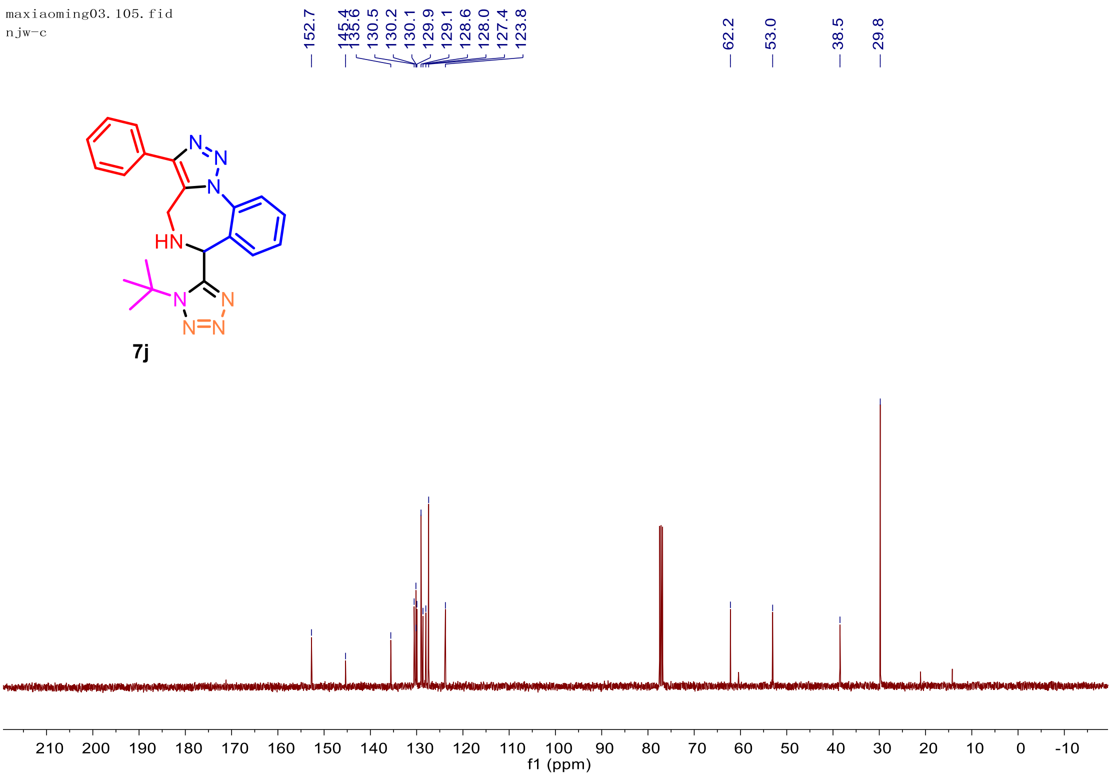

^{13}C NMR spectrum of compound 7j (101 MHz, $CDCl_3$)

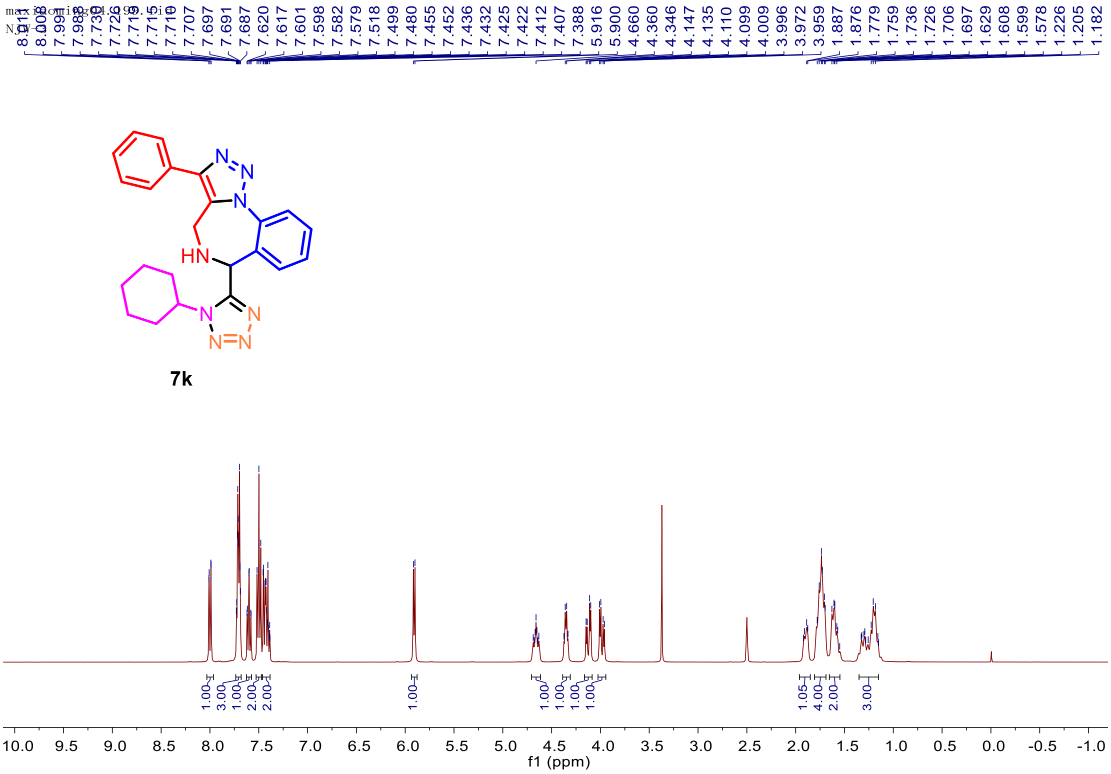

^{1}H NMR spectrum of compound 7k (400 MHz, DMSO-*d$_6$*)

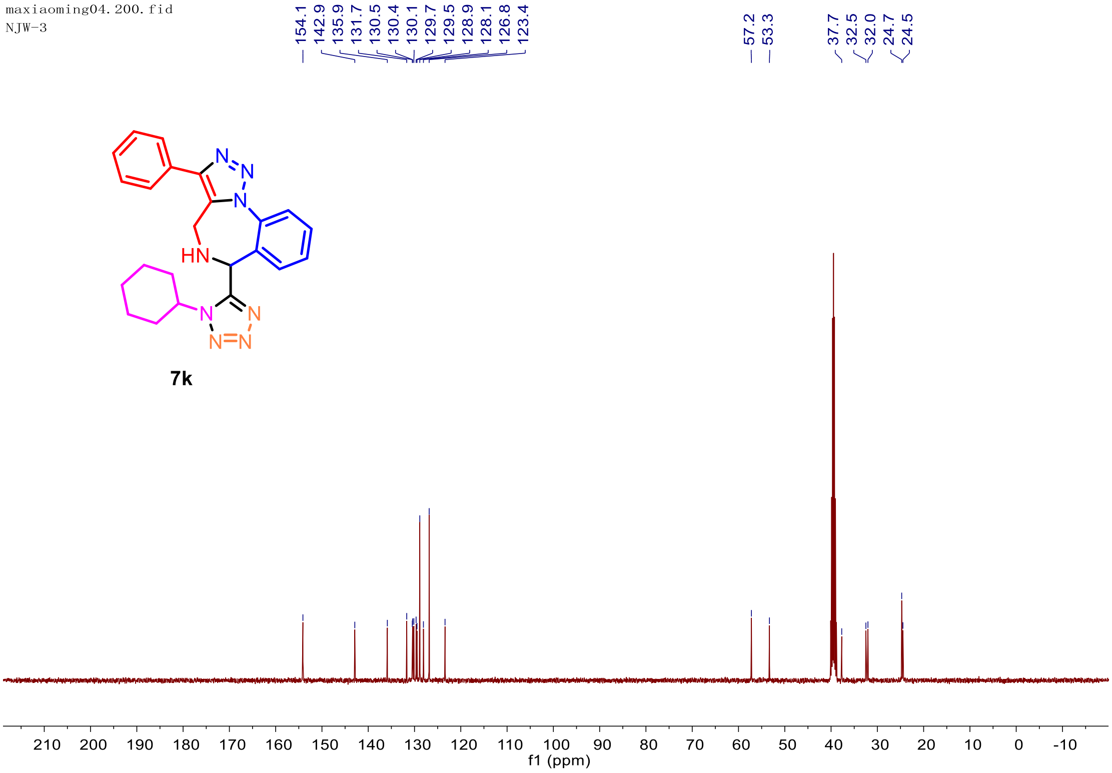

^{13}C NMR spectrum of compound 7k (101 MHz, DMSO-*d$_6$*)

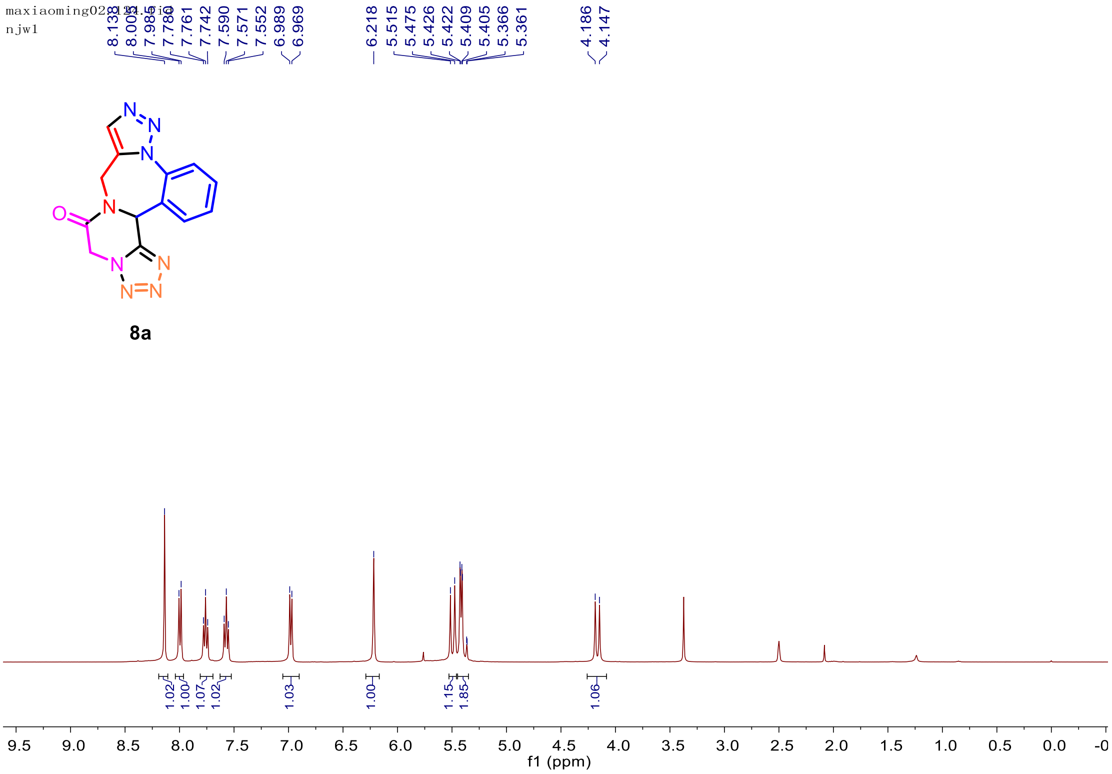

^{1}H NMR spectrum of compound 8a (400 MHz, DMSO-d_6)

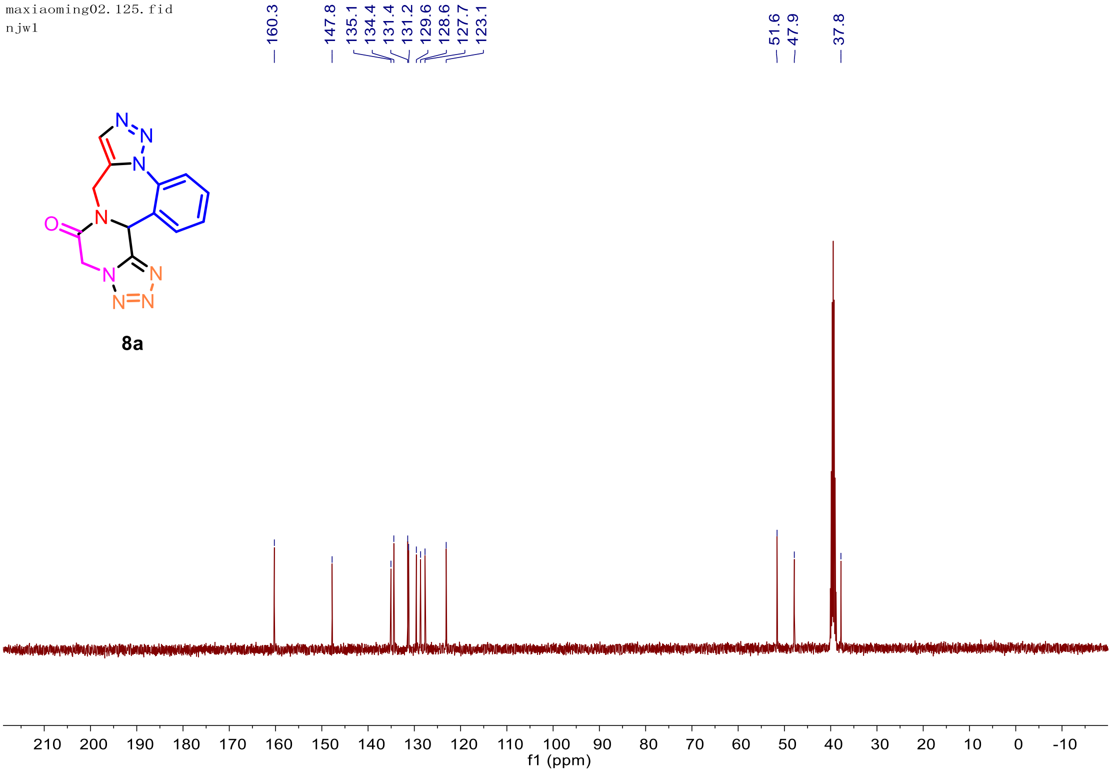

^{13}C NMR spectrum of compound 8a (101 MHz, DMSO-d_6)

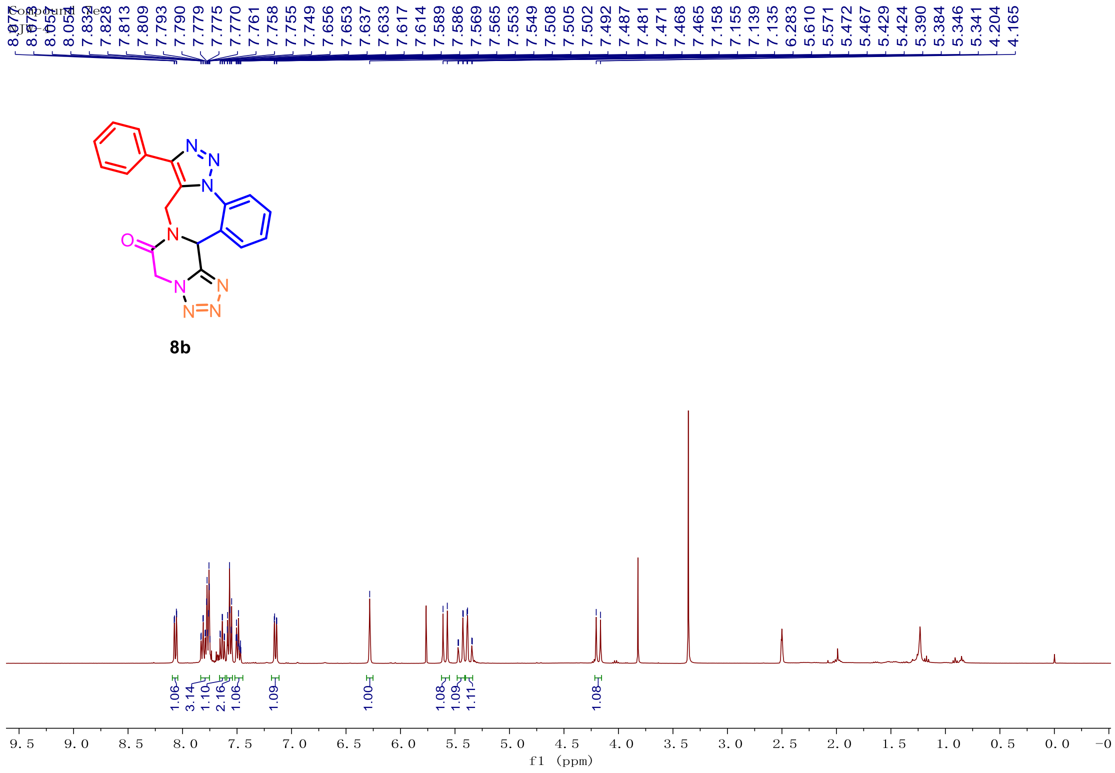

^{1}H NMR spectrum of compound 8b (400 MHz, DMSO-d_6)

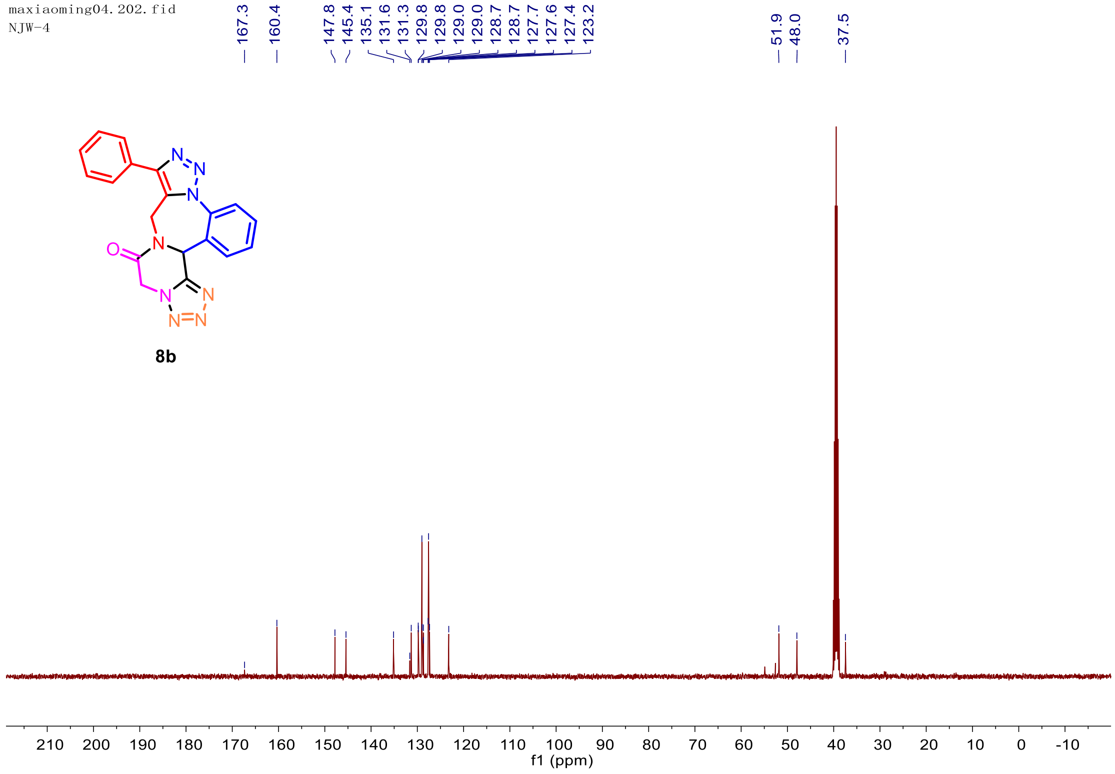

^{13}C NMR spectrum of compound 8b (101 MHz, DMSO-d_6)

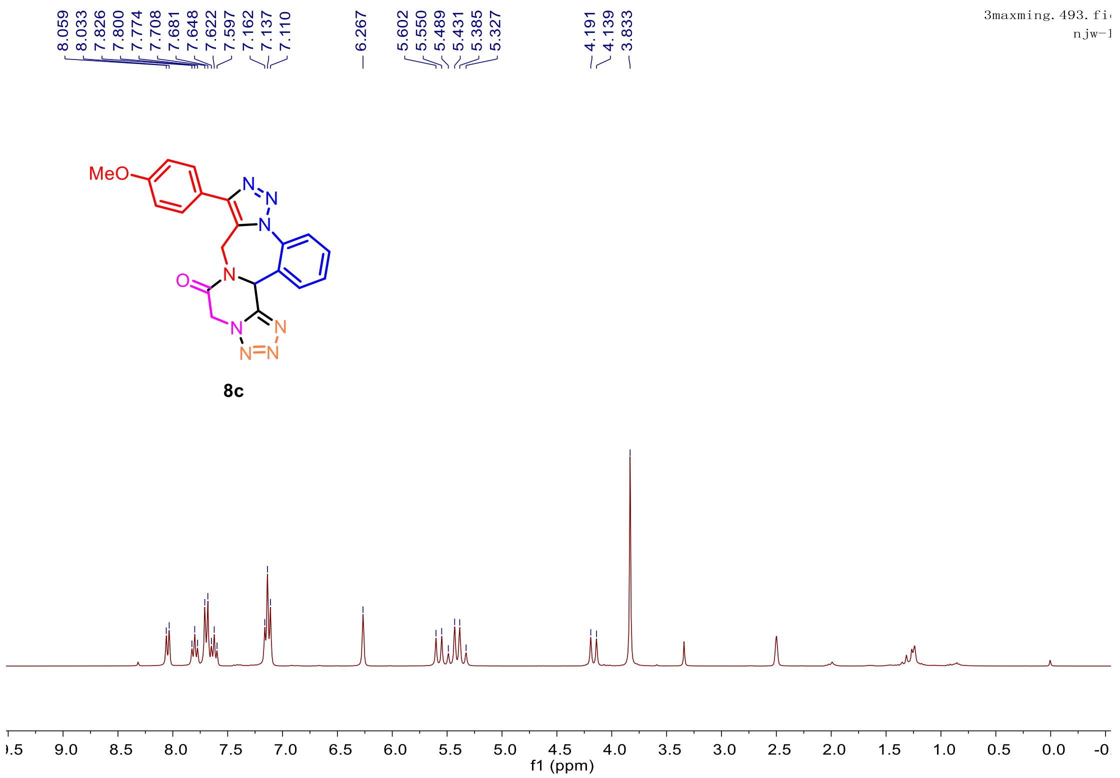

^{1}H NMR spectrum of compound 8c (300 MHz, DMSO-*d6*)

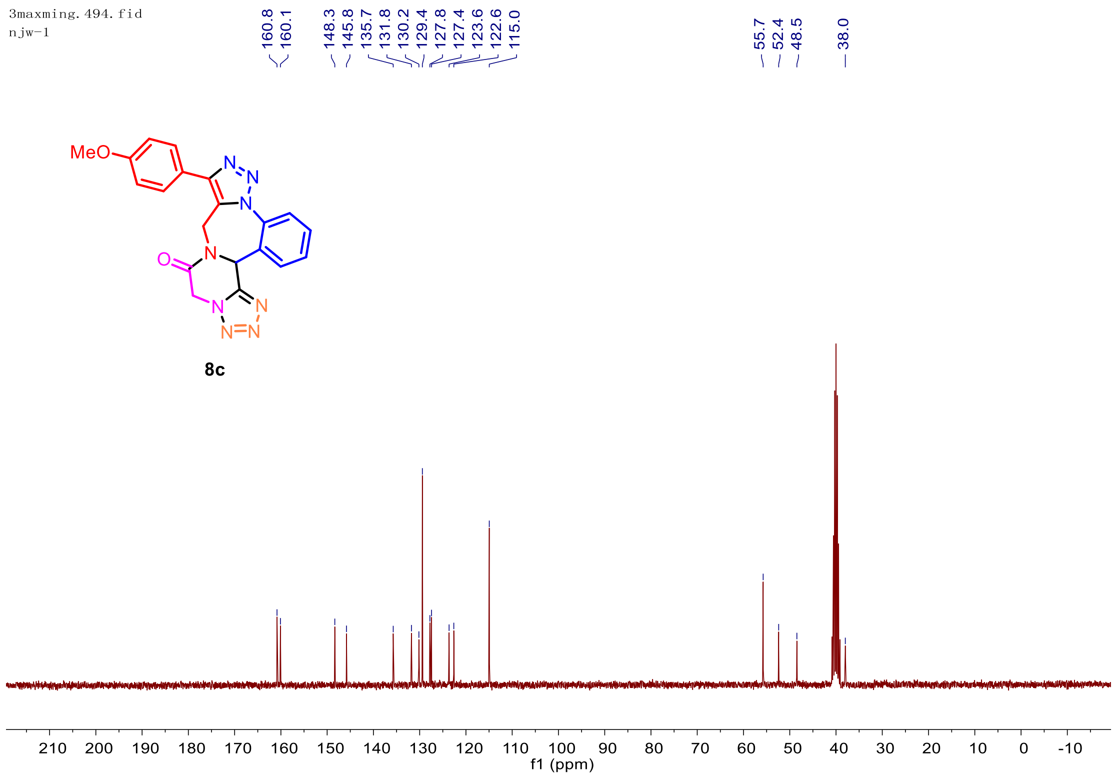

^{13}C NMR spectrum of compound 8c (75 MHz, DMSO-*d6*)

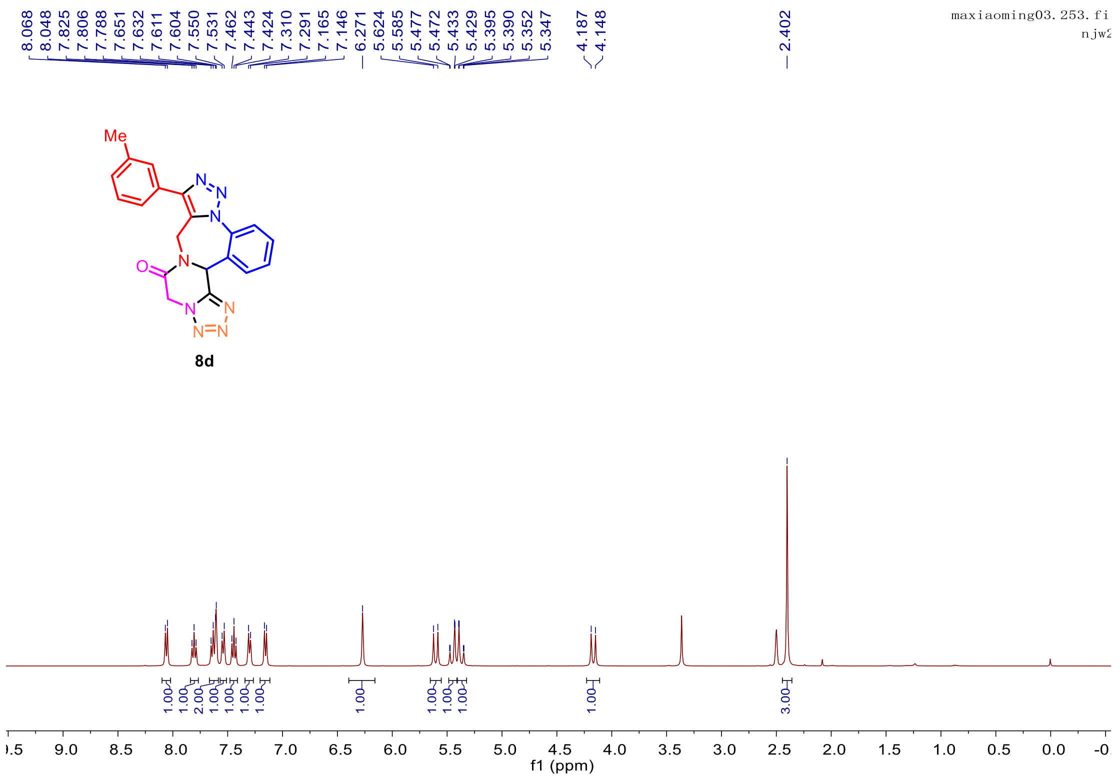

^{1}H NMR spectrum of compound 8d (400 MHz, DMSO-d_6)

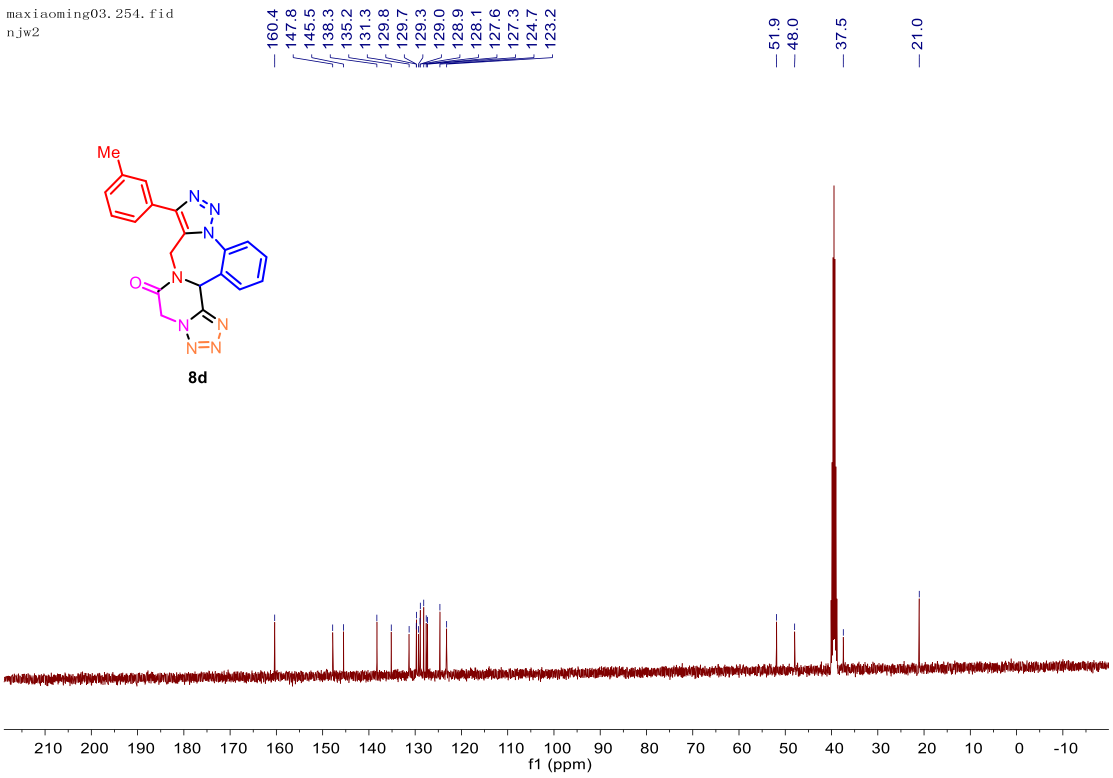

^{13}C NMR spectrum of compound 8d (101 MHz, DMSO-d_6)

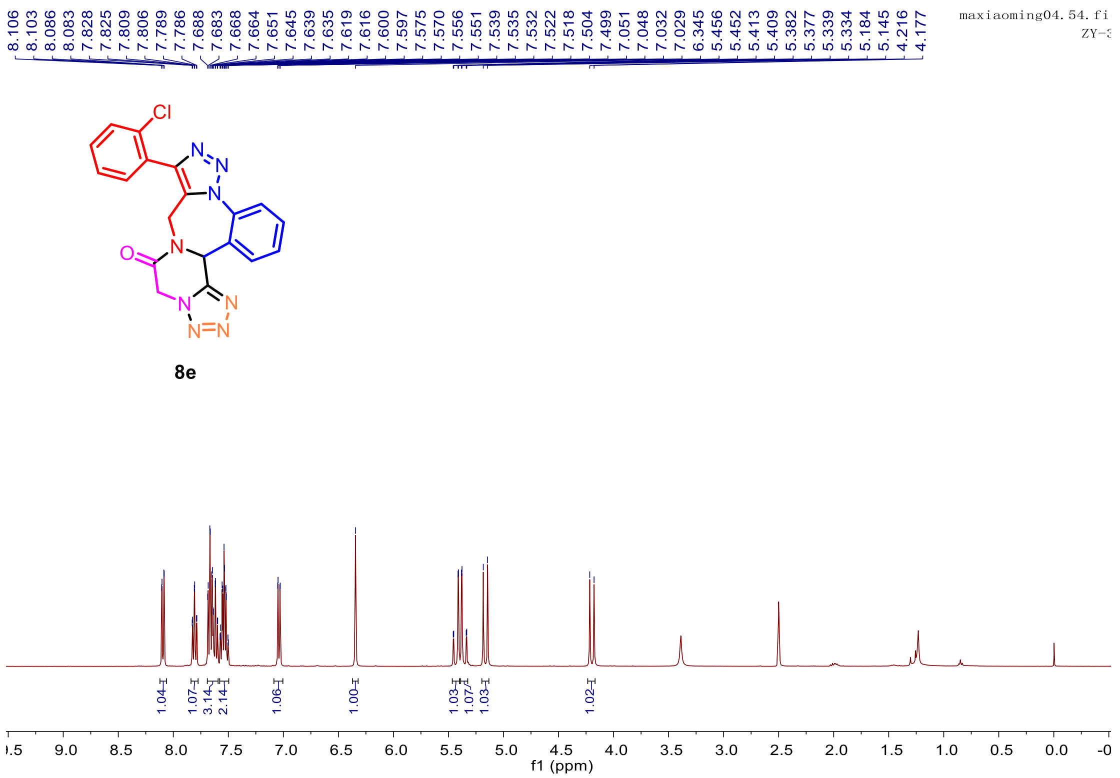

^{1}H NMR spectrum of compound 8e (400 MHz, DMSO-*d*6)

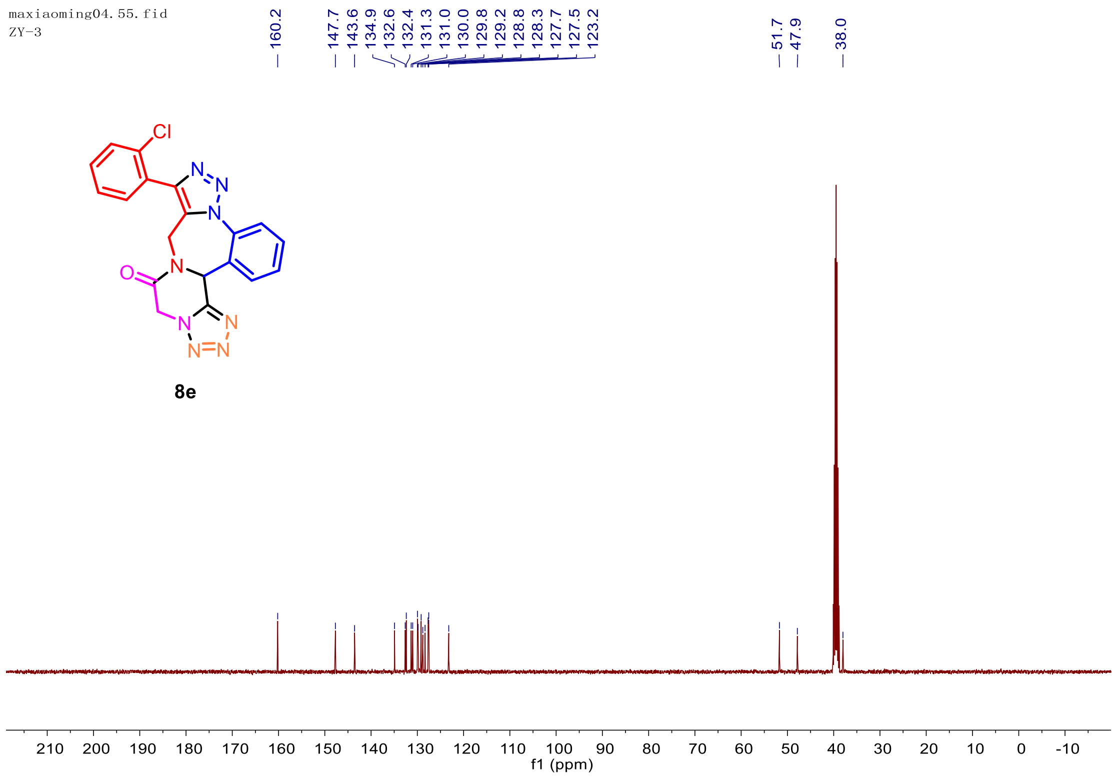

^{13}C NMR spectrum of compound 8e (101 MHz, DMSO-*d*6)

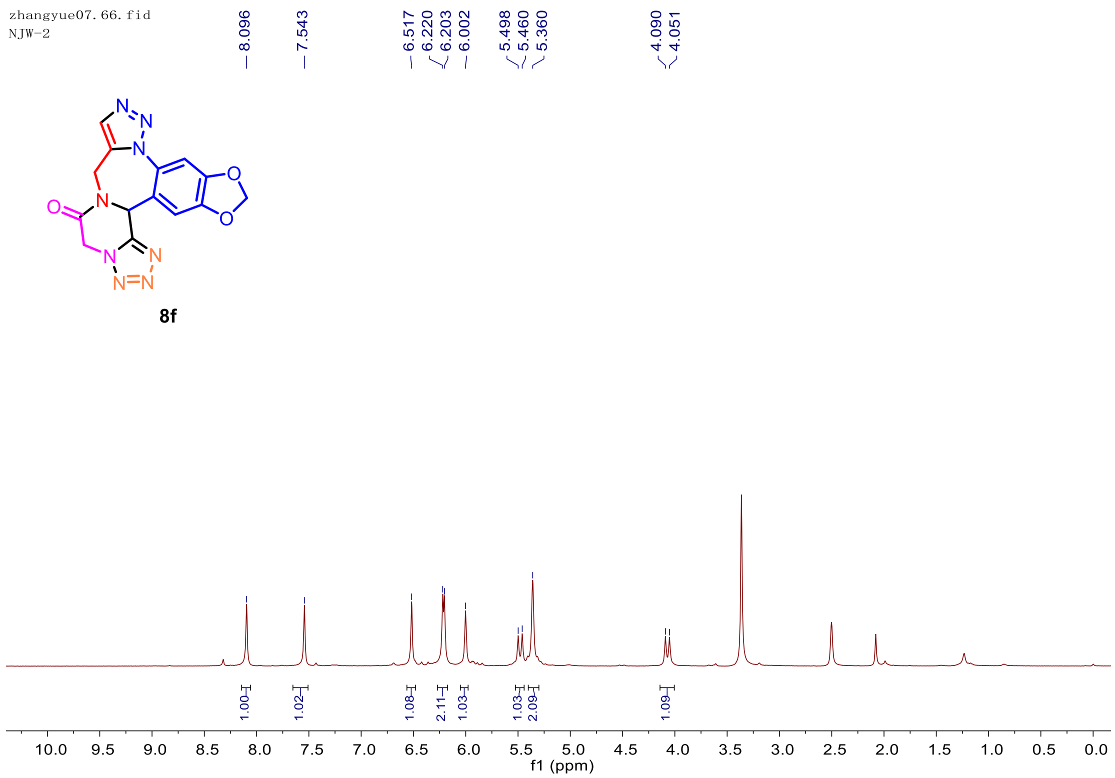

^{1}H NMR spectrum of compound 8f (400 MHz, DMSO-*d*₆)

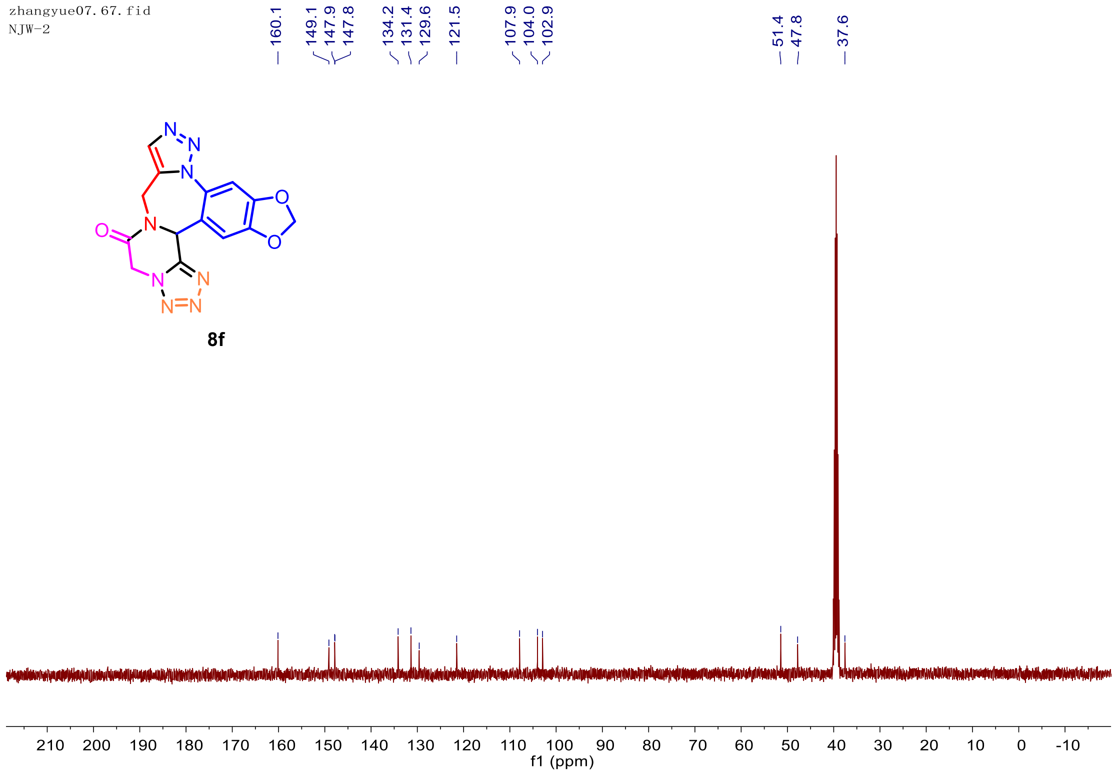

^{13}C NMR spectrum of compound 8f (101 MHz, DMSO-*d*₆)

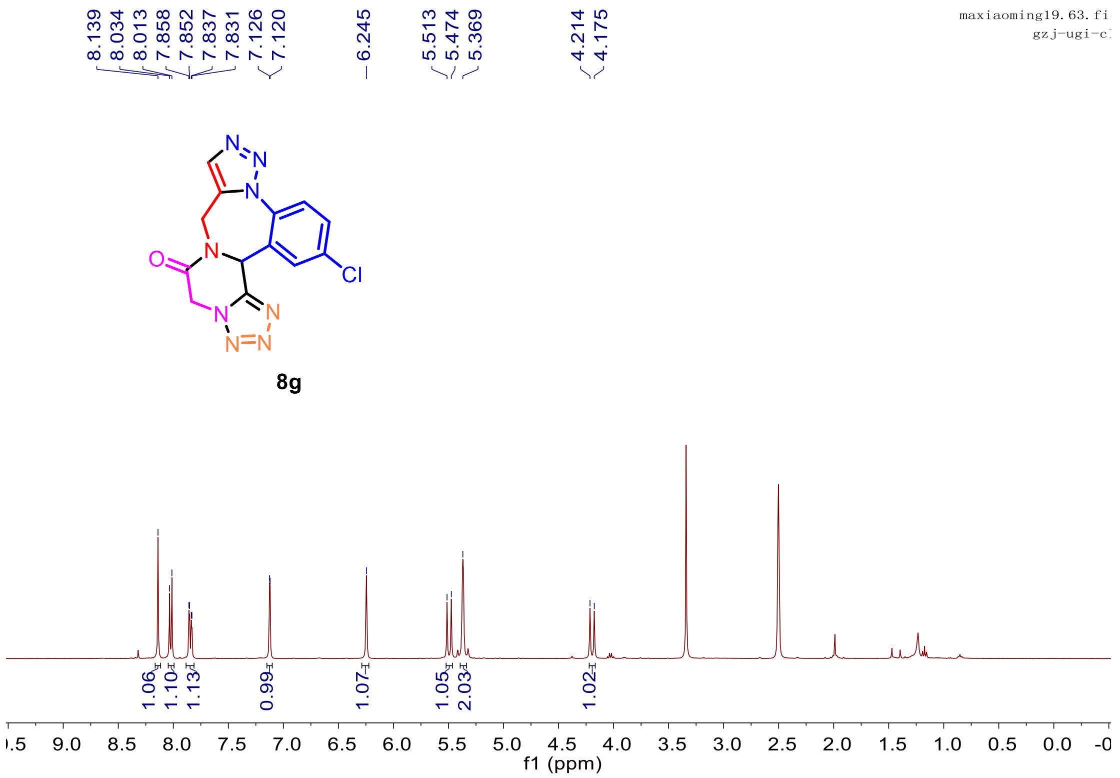

^{1}H NMR spectrum of compound 8g (400 MHz, DMSO-*d*$_6$)

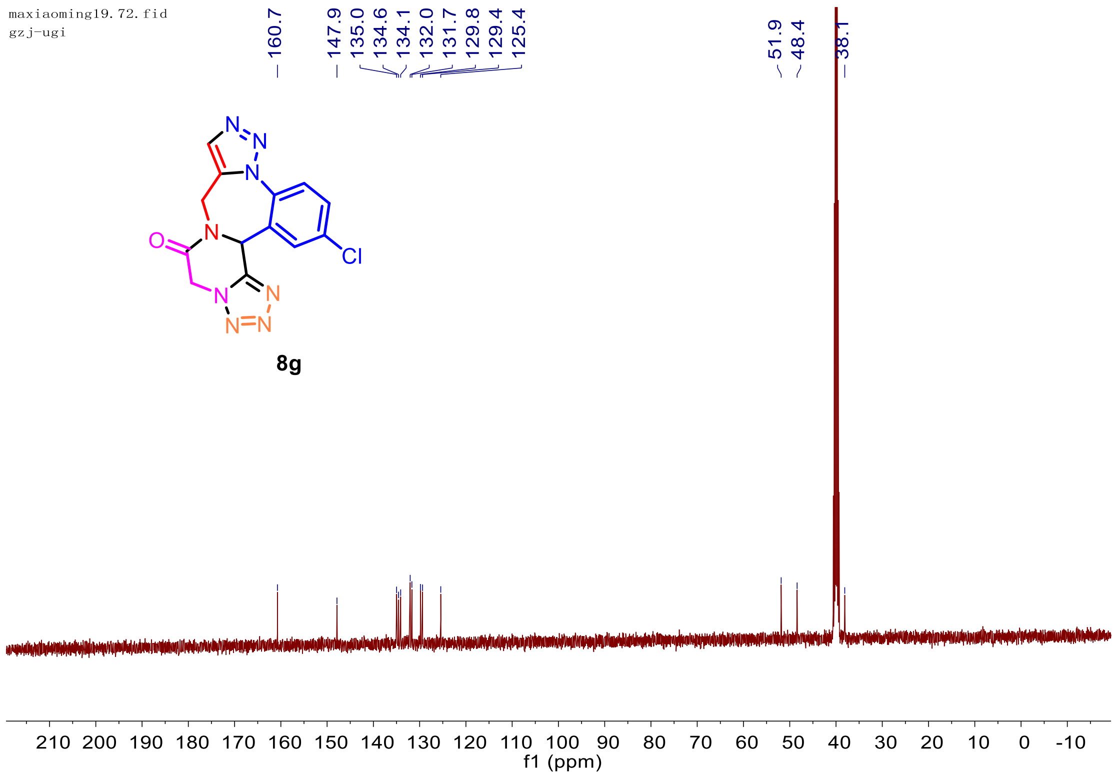

^{13}C NMR spectrum of compound 8g (101 MHz, DMSO-*d*$_6$)

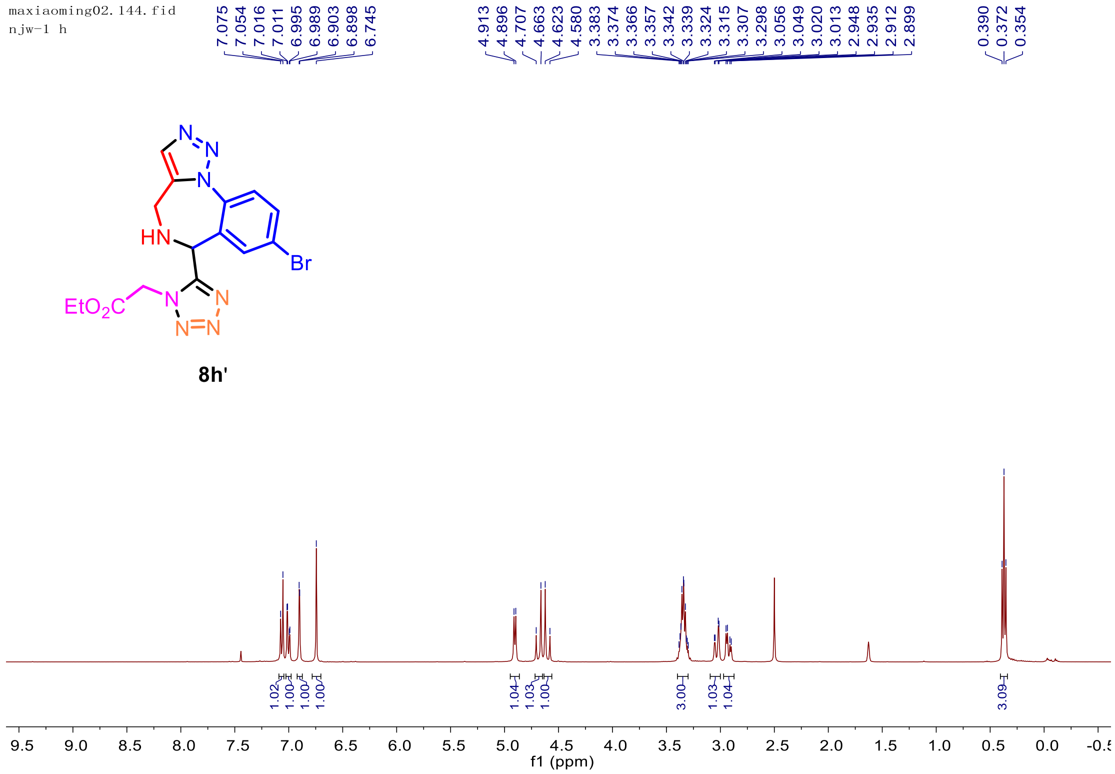

^{1}H NMR spectrum of compound 8h' (400 MHz, DMSO-*d*$_6$)

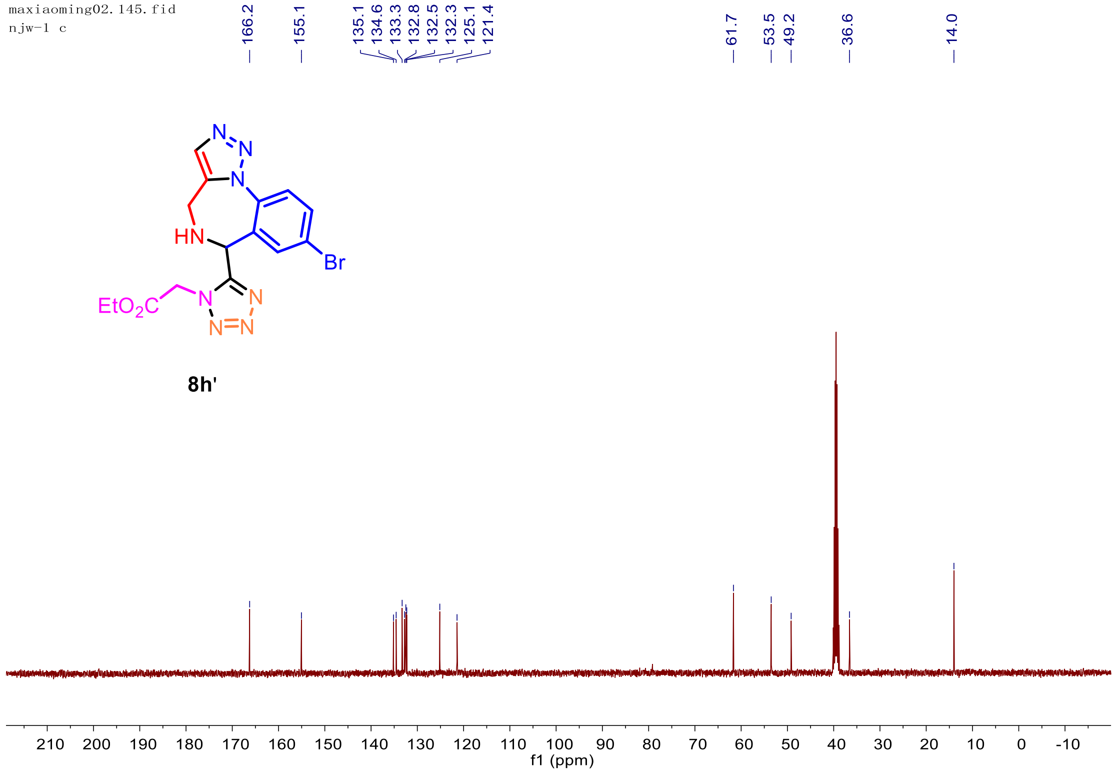

^{13}C NMR spectrum of compound 8h' (101 MHz, DMSO-*d*$_6$)

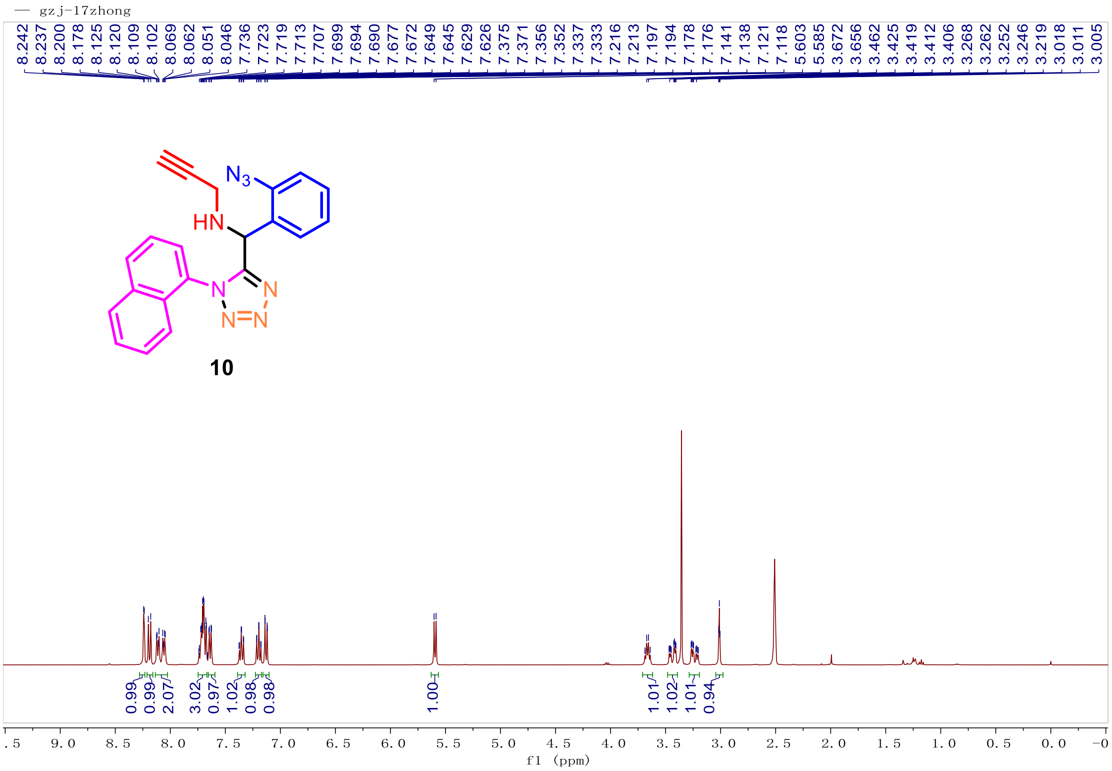

^{1}H NMR spectrum of compound 10 (400 MHz, DMSO-*d6*)

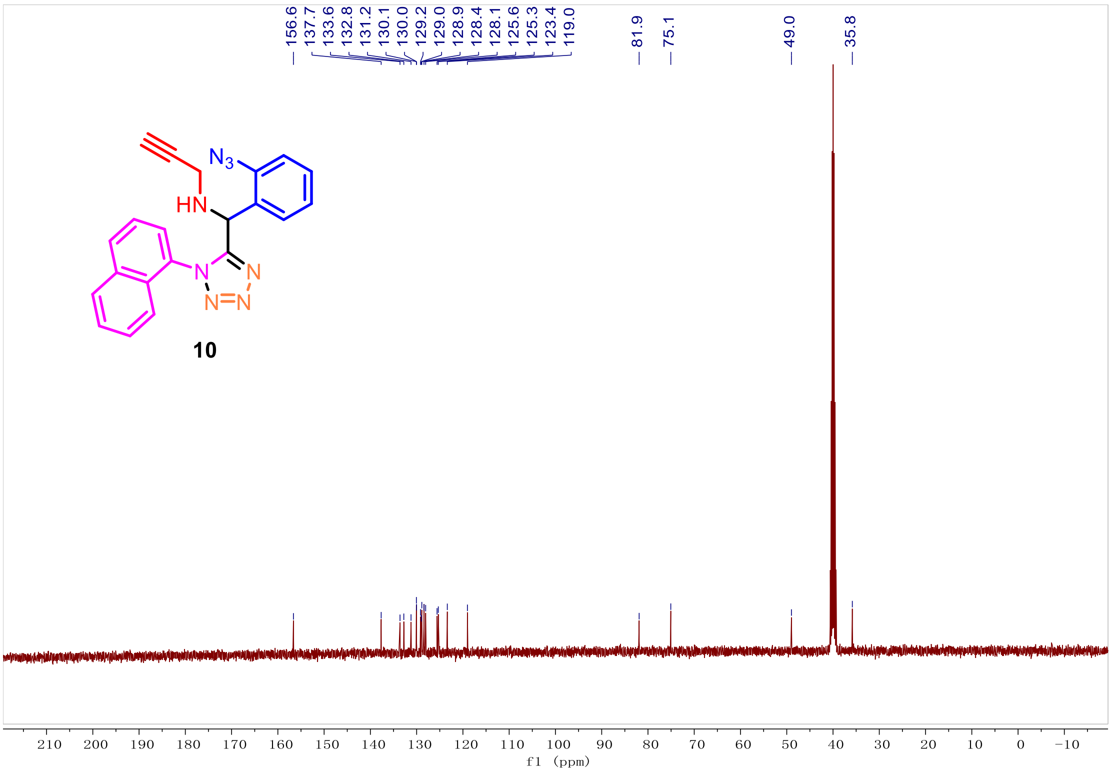

^{13}C NMR spectrum of compound 10 (101 MHz, DMSO-*d6*)

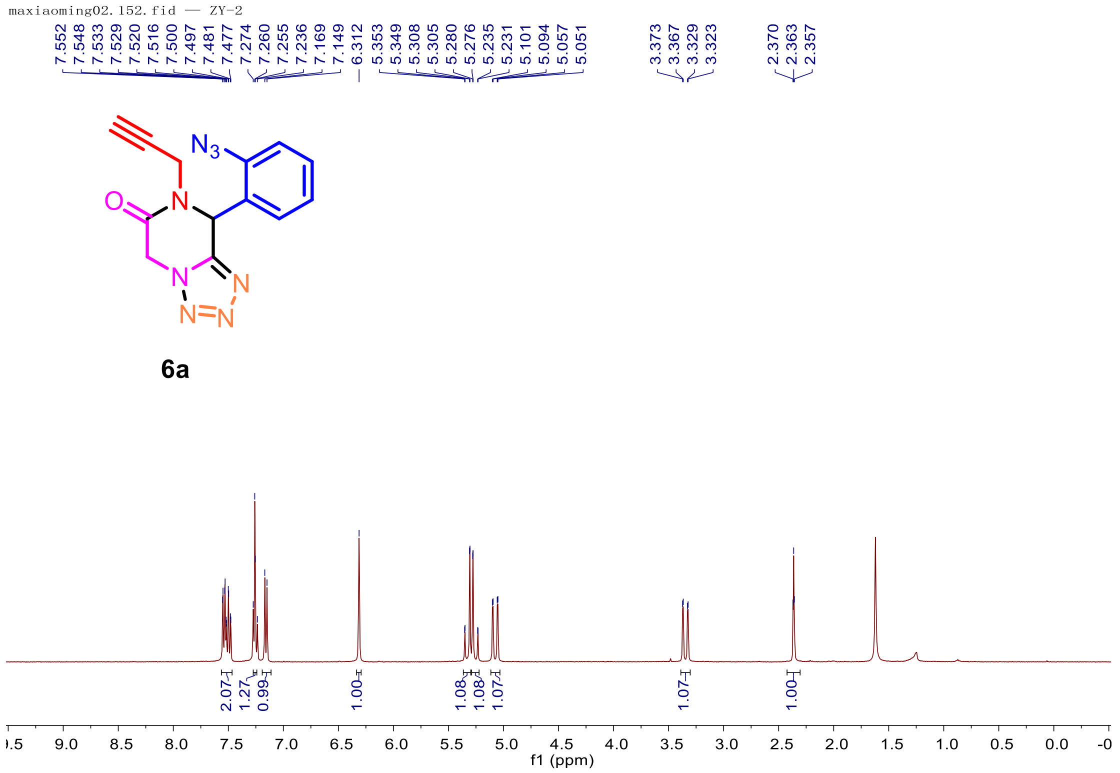

^{1}H NMR spectrum of compound 6a (400 MHz, CDCl3)

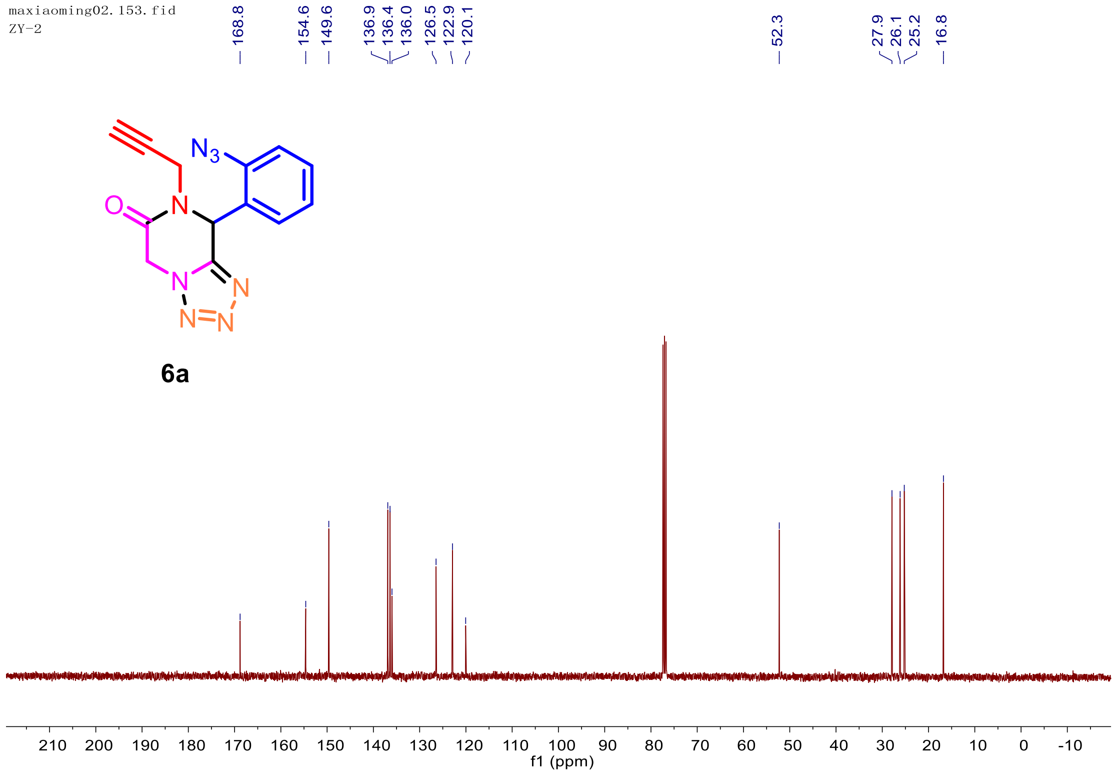

^{13}C NMR spectrum of compound 6a (101 MHz, CDCl3)

7. ^{1}H-^{1}H COSY and HSQC spectra of compound 8a

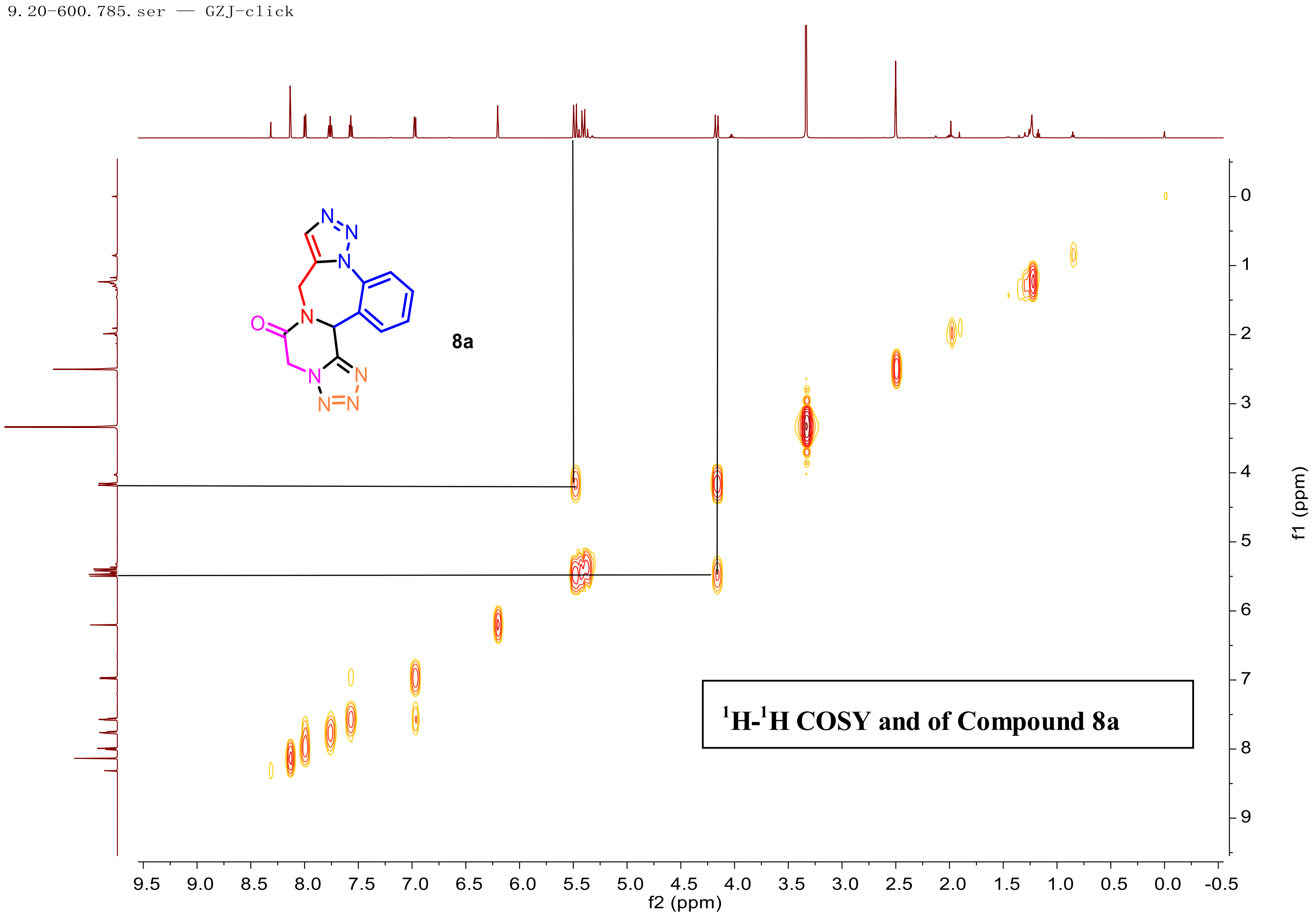

9.20-600.786.ser — GZJ-click

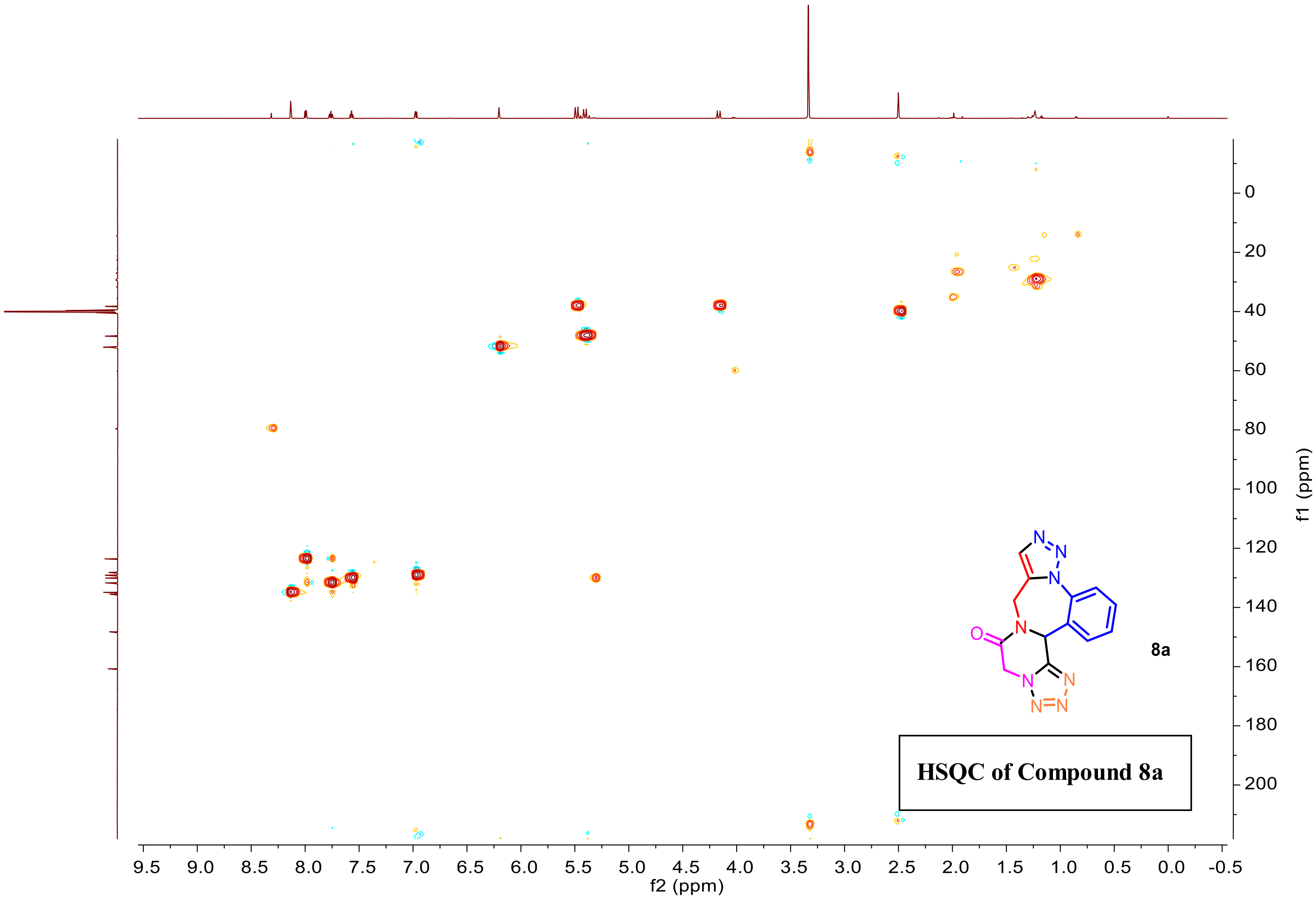

HSQC of Compound 8a
